# Combined heat and salinity stress accelerates oxidative injury and ABA-mediated stress response in cucumber seedlings

**DOI:** 10.3389/fpls.2026.1963962

**Published:** 2026-10-23

**Authors:** Esma Yigider, Melek Ekinci, Sadia Hakeem, Selda Ors, Murat Aydin, Metin Turan, Ertan Yildirim

**Affiliations:** 1 Department of Agricultural Biotechnology, Faculty of Agriculture, Atatürk University, Erzurum, Türkiye; 2 Department of Horticulture, Faculty of Agriculture, Atatürk University, Erzurum, Türkiye; 3 Department of Agricultural Structures and Irrigation, Faculty of Agriculture, Atatürk University, Erzurum, Türkiye; 4 Department of Agricultural Trade and Management, Faculty of Business, Yeditepe University, Istanbul, Türkiye

**Keywords:** abscisic acid, antioxidant, *Cucumis sativus*, metabolic reprogramming, nutrient homeostasis, oxidative stress

## Abstract

**Introduction:**

Cucumber is a heat- and salt-stress-sensitive vegetable crop, whose productivity and marketability are endangered by increasing temperatures and soil salinization driven by climate change. Combined stresses are becoming frequent, yet their interactive effects remain poorly understood.

**Methodology:**

The current study evaluated the negative impacts of high temperature (HT, 40 °C) and salinity (75 and 150 mM NaCl) alone and in combination on cucumber seedlings, under a triplicated completely randomized design. Standard plant growth parameters, micro- and macronutrients, phytohormones, photosynthetic pigment concentrations, oxidative stress biomarkers, osmo-protectants, and antioxidant enzymes were recorded.

**Results:**

Combined HT and 150 mM salt stress caused greater damage to photosynthetic pigments (49-68%), and >98% reduction in growth-promoting hormones, resulting in reduced plant growth (67-93%). Combined stress drastically depleted essential nutrients (94-99.6%) despite lower Na^+^ and Cl^-^ ion concentrations compared to 150 mM NaCl stress alone. Reduced plant growth under combined heat–salinity stress was associated with pronounced disturbances in mineral nutrition and ion homeostasis, although the relative contributions of nutrient imbalance and ionic toxicity could not be distinguished based on tissue mineral concentrations alone. Moreover, plant growth reprogramming toward survival rather than growth was observed with a 206-fold increase in abscisic acid (ABA) and ~99% decline in indole acetic acid, gibberellic acid, and zeatin. Intensified oxidative stress stimulated coordinated activation of ROS (reactive oxygen species) scavenging enzymes, and glutathione-dependent redox metabolism (glucose-6-phosphate dehydrogenase, glutathione reductase, 6-phosphogluconate dehydrogenase, and glutathione-S-transferase increased by 36-, 38-, 41- and 54-fold, respectively), but these remained insufficient to prevent oxidative damage. However, HT and salinity stress triggered distinct pathways: HT preferentially promoted hydrogen peroxide accumulation and activated antioxidant defenses, while salt stress caused greater damage through membrane lipid peroxidation along with ionic imbalance.

**Conclusion:**

These findings suggest that salinity primarily caused physiological impairment, whereas HT amplified oxidative signaling and antioxidant metabolism, providing new insights and potential targets for developing cucumber cultivars with enhanced tolerance to combined stresses. Future research should integrate multi-omics technologies with functional genomics to study the molecular networks regulating nutrient homeostasis, antioxidant metabolism, and ABA signaling under combined stress.

## 1 Introduction

Climate change has increased the frequency of extreme temperature and rainfall events (Seneviratne et al., 2023). These climatic conditions interact with harsh soil conditions like salinity and nutrient deficit, exposing plants to multiple stresses simultaneously (Hassani et al., 2020). Inherently, plants can cope with individual stresses by regulating their morpho-physiological functions and redox homeostasis (Rivero et al., 2022). However, when plants encounter compound stresses, they not only affect plant growth and productivity but also interactions with other organisms, thereby affecting the overall sustainability of the agricultural production system (Zandalinas et al., 2021). Therefore, it is crucial to understand the interaction of different stresses and their combinational effect on plant survival and growth.

Soil salinity is a major abiotic stress not only because of its impacts but also due to its intensifying frequency of occurrence (Ayoub et al., 2021). Salt stress affects ~1 billion ha of land, spanning over 50% of the irrigated land (Basu et al., 2021). It retards plant growth and development due to oxidative stress accompanying physiological drought, adding osmotic pressure as well (Ayoub et al., 2021). Under saline soil or brackish water conditions, plants excessively absorb harmful ions such as Na^+^ and Cl^−^, that interferes with metabolic processes and reduces plant productivity (Waqas et al., 2025). This stress situation is further exacerbated by climate-driven high temperature waves. High-temperature stress has been intensifying, with hottest nights warming faster at 0.32 °C per decade, and compound stress events are more severe, frequent, and prolonged (Emerton et al., 2026). Heat stress damages chloroplast structure in plants and causes irreversible oxidative stress injury due to extreme build-up of reactive oxygen species (Pucciariello et al., 2012).

Various stress adaptation mechanisms have been reported. For instance, under drought and/or salinity stress, stomata are closed to avoid dehydration, while under heat stress, plants generally open their stomata and increase transpiration to cool the canopies (Hsu et al., 2021). However, when drought/salinity and heat stress occur simultaneously, stomata are typically closed. Combined stress (heat and salinity) significantly reduces photosynthesis by affecting carbon assimilation, thus leading to reduced productivity (Rodrigues et al., 2024). This response, however, depends on the timing and intensity of the stress (Zandalinas et al., 2024). For instance, at low salinity levels, stomatal conductance was unaffected, whereas combined high temperature and high salinity significantly reduced stomatal conductance and induced ROS accumulation (Li et al., 2024a). Some studies reported that under combined heat and salinity stress, heat stress was the dominant factor that intensified the stress response in tomato (Li et al., 2024b). Contrastingly, it has also been reported that combined heat and salinity stress may alleviate the damage caused by salinity stress. This was explained by the accumulation of osmotic regulation caused by higher K^+^ concentration and a lower Na^+^/K^+^ ratio under compound stress (Rivero et al., 2014). Thus, there is a need to evaluate the individual response of economically important crops, especially salt- and heat-sensitive crops like vegetables, to elucidate the adaptation mechanisms.

Cucurbits, including cucumber (*Cucumis sativus* L.), are among the most vulnerable crops to climate change (Sun et al., 2026). Cucumber is rich in antioxidants, lycopene, and vitamins (A and C), and magnesium (Rashidi et al., 2024), and has health benefits, including lowering cancer risk. Economically, it is an important crop grown over an area of 2.29 million hectares and with 87.84 million tons of production in 2024 (FAOSTAT, 2026). China is the largest producer with 68.63 million tons, followed by Turkey with 1.74 million tons (FAOSTAT, 2026). Despite increasing demand, cucumber yield and quality are decreasing due to escalating abiotic stresses, especially high temperature and salinity (Kaur and Sharma, 2022). Cucumber requires an optimum temperature of 20 °C to 30 °C. However, climate-driven global warming exposes the crop to high temperature frequently. Rising temperatures beyond 35 °C disrupt the physiological structure of flowers, affecting male and female inflorescences and fruit shape (Olarewaju et al., 2023). It also reduces leaf area, leading to poor seedling vigor, particularly through inhibition of protein synthesis and several biological metabolisms (Shalaby et al., 2022). Moreover, total chlorophyll content was also reported to be reduced under high temperature stress (Ergin et al., 2021). As a salt-sensitive crop, cucumber productivity is threatened by increasing soil salinization (Sun et al., 2026). Salt stress reduces growth through four interconnected pathways: first, an osmotic phase followed by Na and Cl ions toxicity, leading to disruption of nutrient homeostasis, and finally oxidative stress damage (Abbas et al., 2023). Injury from salt stress is stage-dependent, with germination and the seedling stage being the most vulnerable to ionic and osmotic disruption (Sun et al., 2026). Thus, it is important to understand cucumber seedling responses to salt stress during initial growth stages to improve germination and seedling establishment.

Although substantial research has examined the effects of heat and salinity stress separately in cucumber, little is known about their combined effects on cucumber seedlings. This study hypothesized that combined heat and salt stress intensifies the effects of individual stresses on cucumber seedlings, resulting in reduced growth, decreased concentrations of photosynthetic parameters, disruption of nutrient homeostasis, oxidative stress, and hormonal remodulation. The research aimed to: (i) explore the effects of high-temperature stress (40 °C), salt stress (low – 75 mM and high – 150 mM), and the combination of stresses on seedling growth and biomass, and (ii) evaluate their impacts on photosynthetic traits, physiological and biochemical stress markers, and the response of antioxidant and growth hormones. Cucumber seedlings were subjected to high-temperature and/or salt stress. Plant growth and biomass parameters, stress-responsive traits, growth hormones, and antioxidant enzyme concentrations were recorded.

## 2 Methodology

### 2.1 Plant material and growth conditions

The research was conducted under semi-controlled greenhouse conditions. The temperature and humidity of the greenhouse were controlled by a sensor, with the temperature averaging 25 (± 2) °C during the day and 18 (± 2) °C at night, and the humidity level consistently maintained at around 60% (± 5). Cucumber (*Cucumis sativus* L. cv. Oguzcan 8 F1) seeds were sown in seedling trays filled with peat. Approximately 30 days later, when the seedlings reached planting size, they were transferred to 1.8L pots, one seedling per pot. The pots were prepared by mixing garden soil, peat, and perlite in a 2:1:1 ratio by volume.

The water requirement of the plants was monitored throughout the experiment. The amount of water supplied to the pots was determined every 2–3 days in the morning based on the water requirement measured in fully watered control pots using a portable hygrometer (HH2 Hygrometer, WET Sensor, Delta-T Devices, Cambridge, England). The same irrigation approach was maintained during the high-temperature treatment, and no additional irrigation was applied specifically because of the increased temperature. Thus, although the higher temperature may have increased evaporative demand, the experimental design did not intentionally impose an additional drought treatment. This procedure was used to maintain comparable water management among treatments. Salinity treatments (75 and 150 mM NaCl) were initiated 3 days after transplanting. After approximately 15 days of salinity exposure, when clear salinity-induced stress symptoms and growth inhibition were observed, we imposed the high-temperature treatment at 40/30 °C (day/night) for 3 days.

The experiment used a randomized complete block design with 3 replications, each containing 6 plants. The study used 7 treatments: control, 75 mM salt, 150 mM salt, high temperature (40/30 °C), high temperature + 75 mM salt, and high temperature + 150 mM salt. Data were recorded for a total of 126 plants. The initial substrate EC was approximately 1–2 dS m^-1^, and it increased progressively with NaCl concentration, reaching approximately 8–10 dS m^-1^ under 75 mM NaCl and 15–18 dS m^-1^ under 150 mM NaCl at the end of the experiment.

### 2.2 Data recording for growth parameters

Standard seedling growth and biomass parameters included stem diameter (SD, mm), plant height (PH, cm), fresh and dry weights (g), i.e., shoot fresh weight (SFW), root fresh weight (RFW), shoot dry weight (SDW), and root dry weight (RDW). The PH was measured using a scale, from base to the last growing tip of the seedling. The SD was measured using a vernier caliper (500-196-30, Mitutoyo America Corporation, Aurora, Illinois, US). Dry weight was recorded after drying the plants at 60°C for 48 hours.

### 2.3 Nutrient analysis

Macronutrients including nitrogen (N), calcium (Ca), phosphorus (P), magnesium (Mg), potassium (K), and sulfur (S) (measured in percentages), and micronutrients including manganese (Mn), iron (Fe), boron (B), zinc (Zn), sodium (Na), copper (Cu), and chloride (Cl) (measured in mg kg^-1^), were extracted from the oven dried samples using wet digestion method (Ryan et al., 2001; Hakeem et al., 2025). All nutrients were quantified using an atomic absorption spectrophotometer (NovAA400P, Analytik Jena GmbH, Jena, Germany) except N, which was determined using the Kjeldahl method (Bremmer and Mulvaney, 1996).

### 2.4 Determination of plant pigment traits

Plant photosynthetic pigments (expressed as mg g^-1^) including chlorophyll a, b and total chlorophyll, and carotenoids were determined from the fresh leaf. Acetone solution (80%) was used to homogenize samples, and measured at an absorbance of 645 nm, 663 nm and 450 nm using a Multiskan™ GO Microplate Spectrophotometer (Thermo Scientific, Massachusetts, United States) (Ors et al., 2026).

### 2.5 Determination of stress markers and antioxidant enzymes

Oxidative stress biomarkers – malondialdehyde (MDA)and hydrogen peroxide (H_2_O_2_), as well as osmo-protectant proline, and antioxidant enzymes including peroxidase (POD), catalase (CAT), and superoxide dismutase (SOD) glutathione reductase (GR), glutathione S-transferase (GST), and glucose-6-phosphate dehydrogenase (G6PD), 6-phosphogluconate dehydrogenase (6PGDH), and ascorbate peroxidase (APX) were quantified using a Multiskan GO Microplate spectrophotometer (Thermo Scientific, Massachusetts, United States).

Fresh leaf samples were homogenized, and H_2_O_2_ was measured at 390 nm absorbance (Ors et al., 2026), MDA at 532 and 600 nm (Shams et al., 2019), and proline at 520 nm (Bates et al., 1973) and expressed as mmol kg^-1^.

The antioxidant enzymes were determined using fresh leaf samples homogenized in extraction buffer (50 mM phosphate buffer and 1 mM EDTA) following protocols by Ors et al. (2026) and expressed as EU g^-1^ leaf. The CAT activity was determined following the spectrophotometric method (Lück, 1965). Catalase decomposes H_2_O_2,_ which decreases absorbance (Ors et al., 2026) that is measured at 240 nm for one minute (Singla et al., 2019). The POD activity was determined using the guaiacol oxidation method. A reaction involving guaiacol and H_2_O_2_ as substrates results in a color compound that increases absorbance, monitored at 470nm (Angelini et al., 1990). The SOD activity was determined using the nitro blue tetrazolium (NBT) method. One unit was defined as the amount of enzyme required to inhibit 50% of photochemical reduction of NBT (Agarwal and Pandey, 2004).

### 2.6 Determination of plant growth hormones

For hormone analysis, plant samples were purified using the method first described by Kuraishi et al. (1991), and hormone contents were separated using a Zorbax Eclipse-AAA C-18 column on an Agilent 1200 HPLC instrument as described by Turan et al. (2014) and determined by UV detector.

### 2.7 Statistical analysis

All statistical analyses were performed using R version 4.1.2 (Team, 2020). The effects of temperature and salinity were evaluated using a two-way analysis of variance (ANOVA), with temperature (ambient and high temperature) and salinity (0, 75, and 150 mM NaCl) considered as fixed factors. The analysis tested the main effects of temperature and salinity and their interaction (temperature × salinity). The ANOVA was performed (*p<0.001)* using the ‘agricolae’ package (de Mendiburu, 2019). Principal component analysis (PCA) was performed using the packages ‘ggbiplot2’, “FactoMineR” with the scaling and centering features enabled, and PCA biplot was visualized using “factoextra” (Vu, 2011). To assess the correlation among traits, pairwise Pearson’s correlation coefficients (r) were computed using the package ‘corrplot’ (Wei et al., 2021). Means were differentiated based on significance using Tukey’s Honest Significant Difference (HSD) *post hoc* (*p*<0.05) with ‘agricolae’ package. Results were presented as bar graphs generated using packages “agricolae”, “tidyverse” and “ggplot2”. Plots were combined using the “patchwork” package (Pedersen, 2025). To visualize overall clustering patterns among treatments and measured variables, hierarchical clustering analysis (HCA) was performed using standardized (Z-score) data. Euclidean distance was used as the similarity metric, and variables were clustered using Ward’s minimum variance (Ward.D2) linkage method. The clustered heatmap was constructed using the “ComplexHeatmap” package (Gu et al., 2016).

## 3 Results

### 3.1 Effects on plant growth and biomass

Significant differences were found for the growth traits, including plant height (PH) and stem diameter (SD), as well as plant biomass attributes – fresh and dry root and shoot weights (**Supplementary Table S1**; **Figures 1**, **2**). Main and interaction effects were significant for all the traits except for the SD (**Supplementary Table S1**). High temperature (HT) and salinity (75 and 150 mM NaCl) significantly affected all the traits (**Figure 2**). Compared to control (C), salinity reduced SD, SFW, PH, SDW, RFW, and RDW by 47.0, 21.1, 25.0, 58.0, 47.7, and 52.8%, respectively, at 75 mM concentration. Combined stress of 75 mM NaCl and HT also resulted in similar patterns of reduction for all the growth parameters. These reductions were doubled under 150 mM NaCl stress, increasing them to 63, 35, 68, 90, 74, and 81%, respectively (**Figures 1a–f**). High temperature and salinity effects were more pronounced in root traits (**Figures 1d, f**, respectively), causing a reduction of 53-94%.

**Figure 1:**
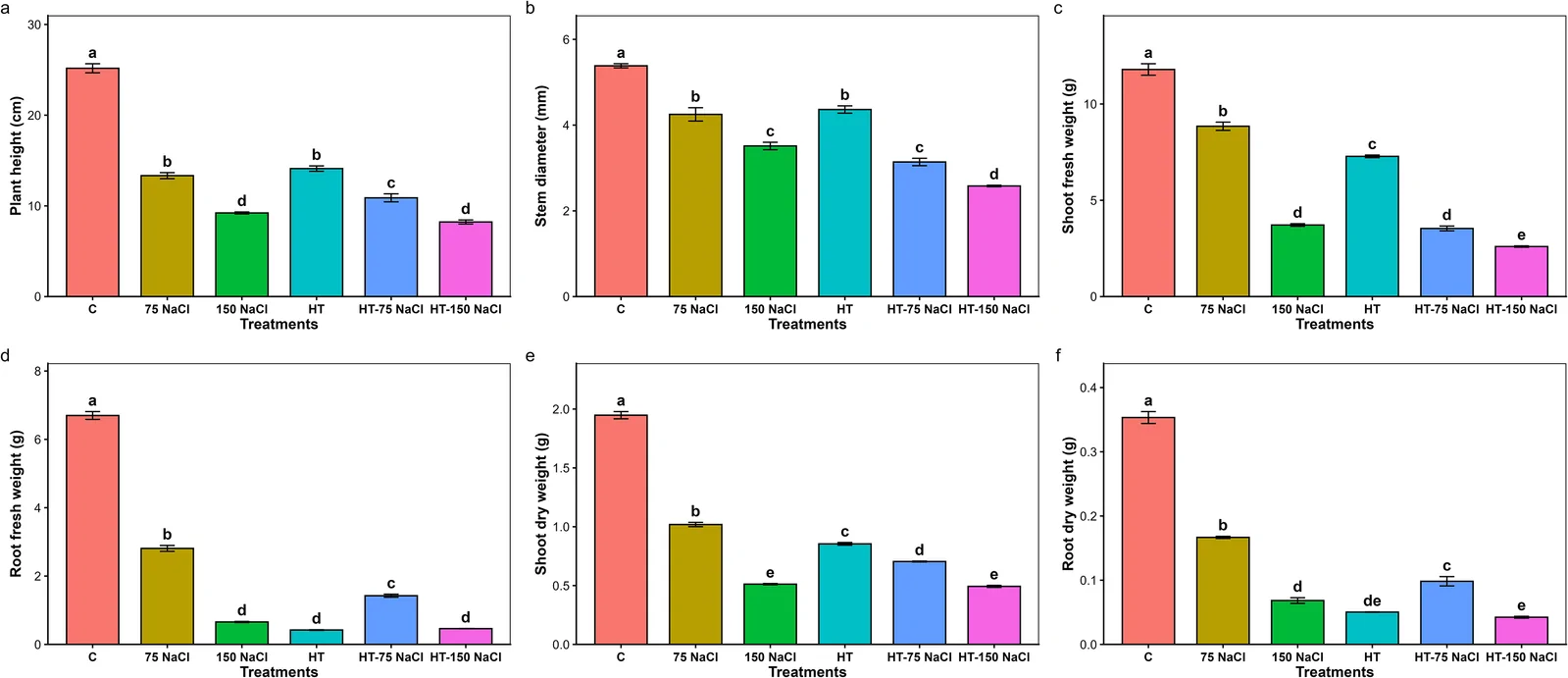
Impact of salinity and heat stress on plant growth attributes of cucumber seedlings, including plant height **(a)**, stem diameter **(b)**, shoot fresh weight **(c)**, root fresh weight **(d)**, shoot dry weight **(e)**, and root dry weight **(f)**. Data is presented as Mean, with standard error represented as error bars. NaCl, sodium chloride; HT, High temperature; C, control. Significant differences are represented by different letters above the bars (*p<0.05*).

**Figure 2:**
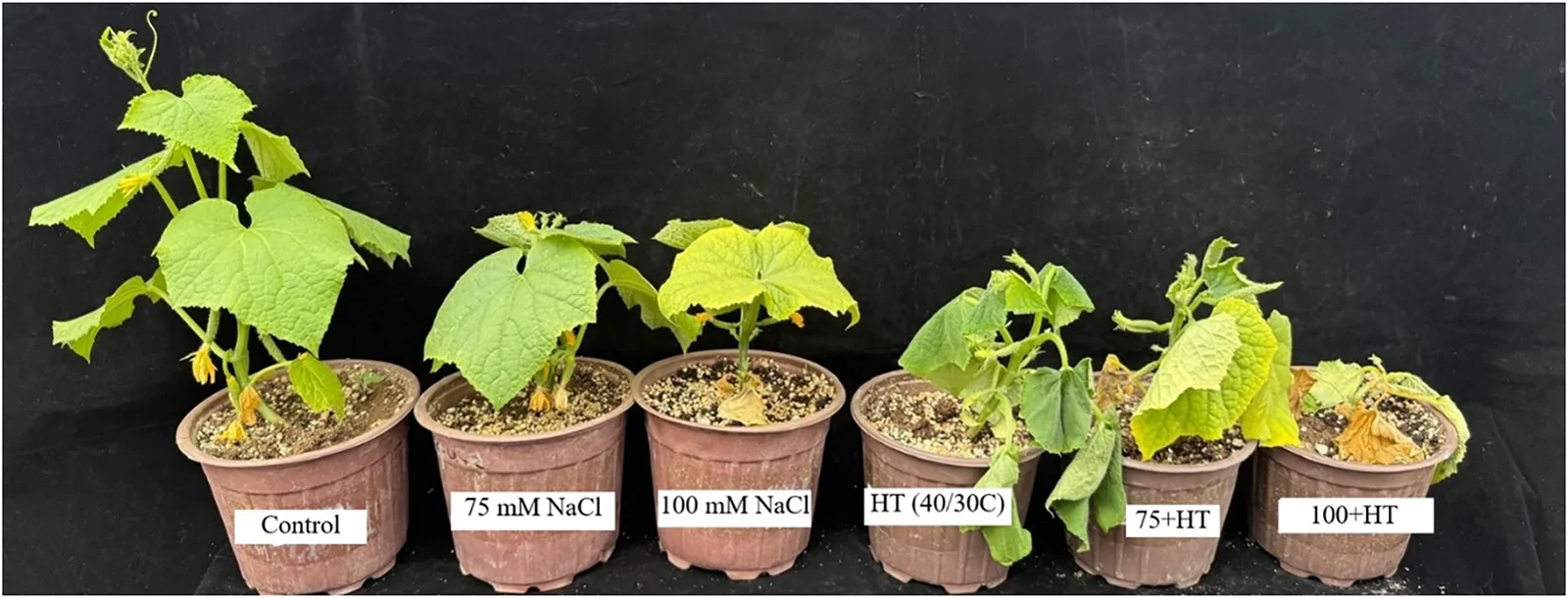
Impact of salinity and heat stress on plant growth attributes of cucumber seedlings. NaCl, sodium chloride; HT, High temperature (40/30°C).

High temperature also reduced the growth parameters, with greater reductions in biomass compared to salinity, up to 38, 94, 56, and 86% reductions in stem and root fresh and dry weights, respectively (**Figures 1c–f**). These effects were further aggravated by combined heat and 150 mM NaCl stress, causing 67, 52, 78, 93, 75, and 88% reductions in PH, SD, SFW, RFW, SDW, and RDW, respectively. Overall, combined treatment of 150 mM NaCl and high temperature had the strongest negative effects on cucumber seedling growth (**Figure 2**).

### 3.2 Effects on pigment traits

Both stresses significantly reduced the carotenoids and chlorophyll concentrations (**Supplementary Table S1**), as indicated by statistical lettering (**Figures 3a–d**).

**Figure 3:**
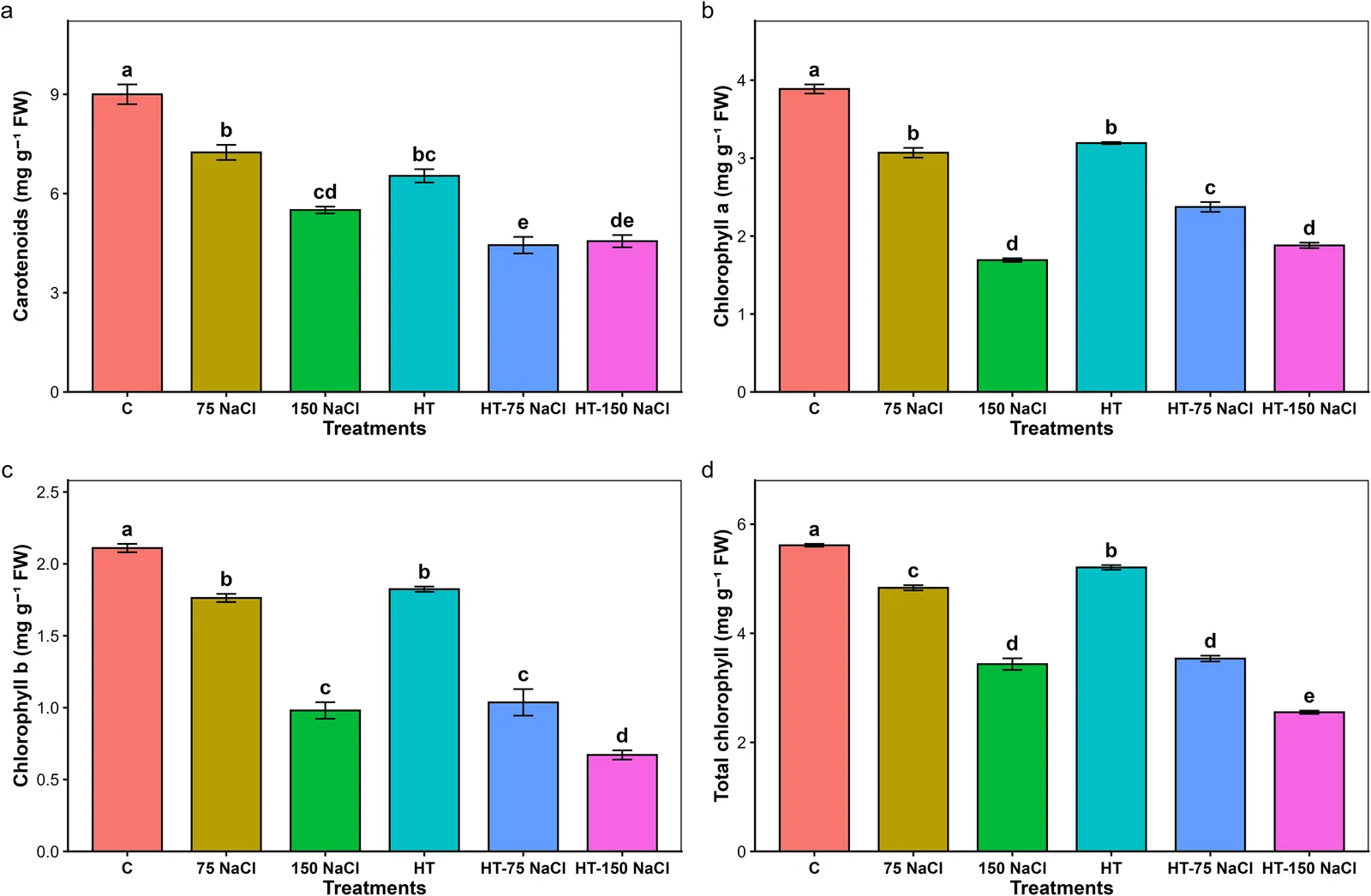
Impact of salinity and heat stress on plant pigment traits in cucumber seedlings, including carotenoids **(a)**, chlorophyll a **(b)**, chlorophyll b **(c)**, and total chlorophyll **(d)**. Data is presented as Mean, with standard errors represented as error bars. NaCl, sodium chloride; HT, High temperature; C, control. Significant differences are represented by different letters above the bars (*p<0.05*).

Salinity stress significantly reduced pigment concentrations, with carotenoids reduced from 9.0 mg g^-1^ to 5.5 mg g^-1^ (-39%) under 150 mM salt stress (**Figure 3a**). Similarly, Chlorophyll a, b, and total chlorophyll reduced up to 1.7, 1.0, and 3.4 mg g^-1^, respectively (-56.5, -53.5, and -38.8%, respectively) under 150mM NaCl stress (**Figures 3b–d**). Although these reductions were less pronounced under 75 mM NaCl stress, causing 21.0, 16.5, and 13.9% reductions.

Heat stress also reduced pigment accumulation; however, the effects were not as drastic as 150 mM NaCl. Carotenoids, chlorophyll a, b, and total chlorophyll were reduced by 27, 18, 14, and 7%, respectively. Combined heat and 75mM salinity stress significantly reduced these pigments by 51, 39, 51, and 37%, respectively. While the impact of combined heat and 150 mM salinity stress was relatively mild, as it restored Chlorophyll a by 11% (**Figure 3b**) compared to 150 mM NaCl stress alone, it reduced only 17, 32, and 26% compared to 150 mM NaCl stress. Overall, 150mM NaCl stress had the most drastic effects on pigment traits.

### 3.3 Effects on plant nutrients

Salinity and heat stress significantly reduced macronutrients (**Figure 4**) and micronutrients (**Figure 5**; **Supplementary Table S1**). Compared to the control, all nutrients were drastically reduced under either stress conditions alone or in combination. However, under combined treatment of both stresses, nutrients were more depleted, causing up to -94% to -99.6% change in all the micro- and macronutrients.

**Figure 4:**
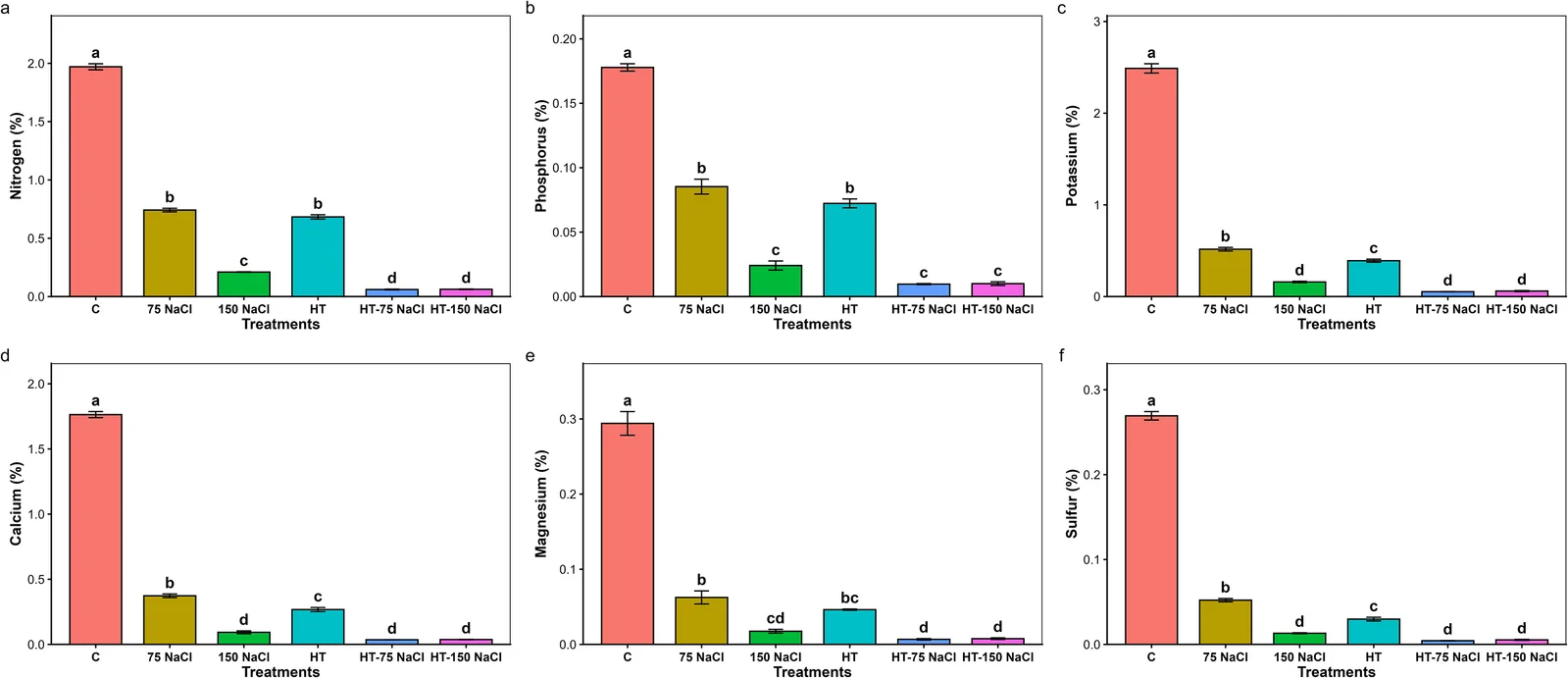
Impact of salinity and heat stress on macronutrients of cucumber seedlings, including nitrogen **(a)**, phosphorus **(b)**, potassium **(c)**, calcium **(d)**, magnesium **(e)**, and sulfur **(f)**. Data are presented as Mean, with standard error represented as error bars. NaCl, sodium chloride; HT, High temperature; C, control. Significant differences are represented by different letters above the bars (*p<0.05*).

**Figure 5:**
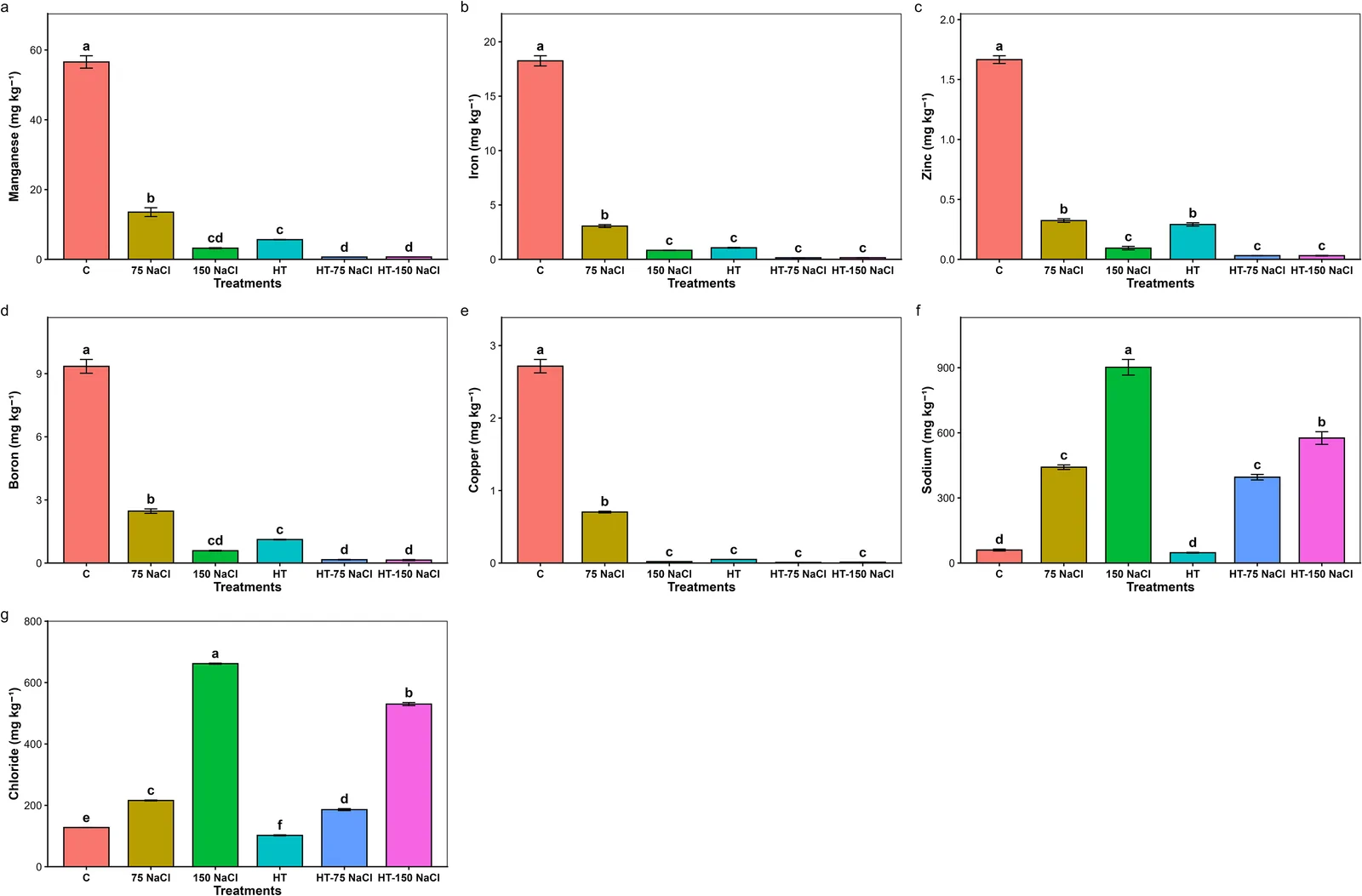
Impact of salinity and heat stress on micro-nutrients and ion concentrations in cucumber seedlings, including manganese **(a)**, iron **(b)**, zinc **(c)**, boron **(d)**, copper **(e)**, sodium **(f)**, and chloride **(g)**. Data is presented as Mean, with standard errors represented as error bars. NaCl, sodium chloride; HT, High temperature; C, control. Significant differences are represented by different letters above the bars (*p<0.05*).

For macro-nutrients, N, P, K, Ca, Mg, and S, 75 mM NaCl stress reduced concentrations up to 62.4, 52.0, 79.3, 78.8, 78.0, and 80.6%, respectively (**Figures 4a–f**). Increasing the salt concentration (150 mM) further reduced the nutrient concentrations by 89.3, 86.4, 93.6, 94.7, 94.0, and 95.1%, respectively. High temperature alone also markedly reduced nutrients by 65, 59, 84, 85, 84, 84, and 89%, respectively. In general, reductions in these nutrient concentrations were comparable to those caused by 75 mM NaCl, but not as drastic as those caused by 150 mM NaCl. Combined exposure to salt and heat stress further aggravated the nutrient depletion. The N, P, K, Ca, Mg, and S were reduced from 2.0, 0.2, 2.5, 1.8, 0.3, and 0.3% under normal conditions to 0.063, 0.010, 0.060, 0.037, 0.008, and 0.005%, respectively under HT + 150 mM NaCl stress. Similar reduction in concentrations was exhibited under 75 mM NaCl stress as well corresponding to 96.9, 94.6, 97.9, 98.0, 97.7, and 98.4% reduction in concentration, respectively.

Micronutrients including manganese (Mn), iron (Fe), zinc (Zn), boron (B) and copper (Cu) were reduced by 76.1, 83.2, 80.6, 73.6 and 74.1%, respectively, under 75 mM NaCl (**Figures 5a–g**). These nutrients were further reduced up to ~95% under 150 mM NaCl stress. High temperature (HT) also markedly reduced micronutrients, causing changes of -94.3% (Fe), -95.4% (Zn), -94.4% (Mn), -93.7% (B), and -99.3% (Cu). Combined application of HT and NaCl stress (both 75 mM and 150 mM) caused similar but drastic reductions of -98.8% (Fe), -99.2% (Zn), -98.2% (Mn), -98.5% (B) and -99.6% (Cu).

In contrast to essential micronutrients, Na and Cl concentrations were significantly increased under salinity stress conditions. However, under HT stress, Na concentrations did not vary significantly compared to the control but were reduced from 59.9 mg kg^-1^ to 441.6 mg kg^-1^ (**Figure 5f**). Moreover, Cl concentrations reduced under HT stress from 127 mg kg^-1^ to 102 mg kg^-1^ (**Figure 5g**). Highest concentrations for these ions were found under 150 mM NaCl stress (902.0 (10-fold) and 661.8 mg kg^-1^ (6-fold), respectively). Under 75 mM NaCl stress, Na and Cl concentrations increased by 5-fold and 2-fold, respectively. Combined stress effects increased Na concentrations comparable to 75mM NaCl, increasing to 395.5 mg kg^-1^ under HT + 75mM NaCl, while 576.0 mg kg^-1^ under HT + 150 mM NaCl. While Cl concentration was lower under HT + 75 mM NaCl (**Figure 5f**), it increased to 530 mM under HT + 150 mM NaCl stress condition (**Figure 5g**).

### 3.4 Effects on oxidative stress markers and osmolyte accumulation

The salinity and heat stress significantly increased the concentrations of stress responsive traits like hydrogen peroxide (H_2_O_2_; **Figure 6a**), malondialdehyde (MDA; **Figure 6b**), proline (**Figure 6c**) and sucrose (**Figure 6d**; **Supplementary Table S1**). Significant differences were found among treatments for all the traits except for 75 mM NaCl and HT for MDA (**Figure 6b**), C and HT, and both salt treatments for proline (**Figure 6c**), C and HT, and combined treatments for sucrose (**Figure 6d**).

**Figure 6:**
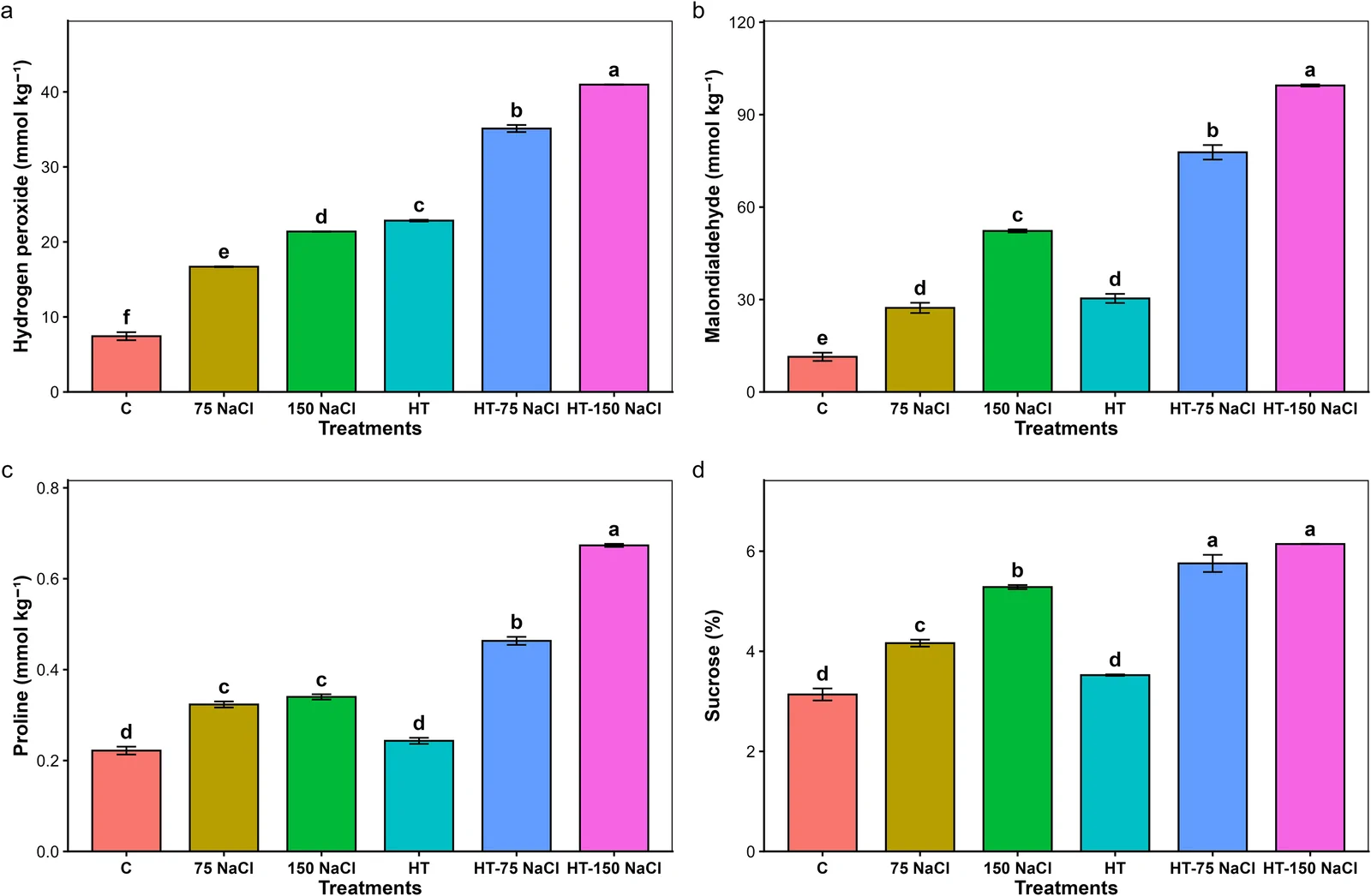
Impact of salinity and high temperature stress on oxidative stress markers and osmolyte accumulation, including hydrogen peroxide **(a)**, malondialdehyde **(b)**, proline **(c)**, and sucrose **(d)**, in cucumber seedlings. Data is presented as Mean, with standard errors represented as error bars. NaCl, sodium chloride; HT, High temperature; C, control. Significant differences are represented by different letters above the bars (*p<0.05*).

The 75 mM NaCl stress increased H_2_O_2_, MDA, proline, and sucrose by 2.3-2.4, 1.5-, and 1.4-fold compared to the control (7.4 mmol kg^-1^), corresponding to 124.5, 139.1, 45.7, and 32.7% increases, respectively. Increasing salt concentration (150 mM) further increased these indices by 187.6, 357.9, 53.2 and 68.4%, respectively. Exposure to HT spiked H_2_O_2_ (+207%) compared to control and salinity stress alone, while proline and sucrose only increased by 10 and 12%, respectively, and hence were not statistically different compared to control. Combined treatment of 75 mM NaCl and HT resulted in an increase of 372, 582 and 109% in H_2_O_2_, MDA and proline, respectively. While 150 mM NaCl and HT increased these indices to 451, 772 and 203%, respectively (**Figures 6a–c**). However, sucrose concentrations remained similar for both combined treatments (84 and 96%, respectively), thus not statistically significant (**Figure 6d**).

### 3.5 Effects on plant growth hormones

Salinity and heat stress significantly reduced all the phytohormones (**Figure 7**; **Supplementary Table S1**) except for abscisic acid, which showed an antagonistic response (**Figure 7b**). Stress conditions significantly reduced the growth hormones. Compared to the control, 75 mM NaCl increased indole acetic acid (IAA), gibberellic acid (GA), salicylic acid (SA), zeatin (ZT), and jasmonic acid (JA) by 86.6, 58.1, 75.9, 81.2, and 85.5%, respectively. Under 150 mM NaCl, the hormones were further reduced up to 95.0-97.9%. Heat stress also reduced the phytohormones by 89% (SA) to 95% (JA). Combined stresses reduced the phytohormone levels to >98%. Contrastingly, compared to control, abscisic acid (ABA) was increased by 1.2-, 5.2-, 10.1-, 36.1- and 206.9-fold under 75 mM NaCl, 150 mM NaCl, HT, HT + 75 mM NaCl and HT + 150 mM NaCl stress, respectively.

**Figure 7:**
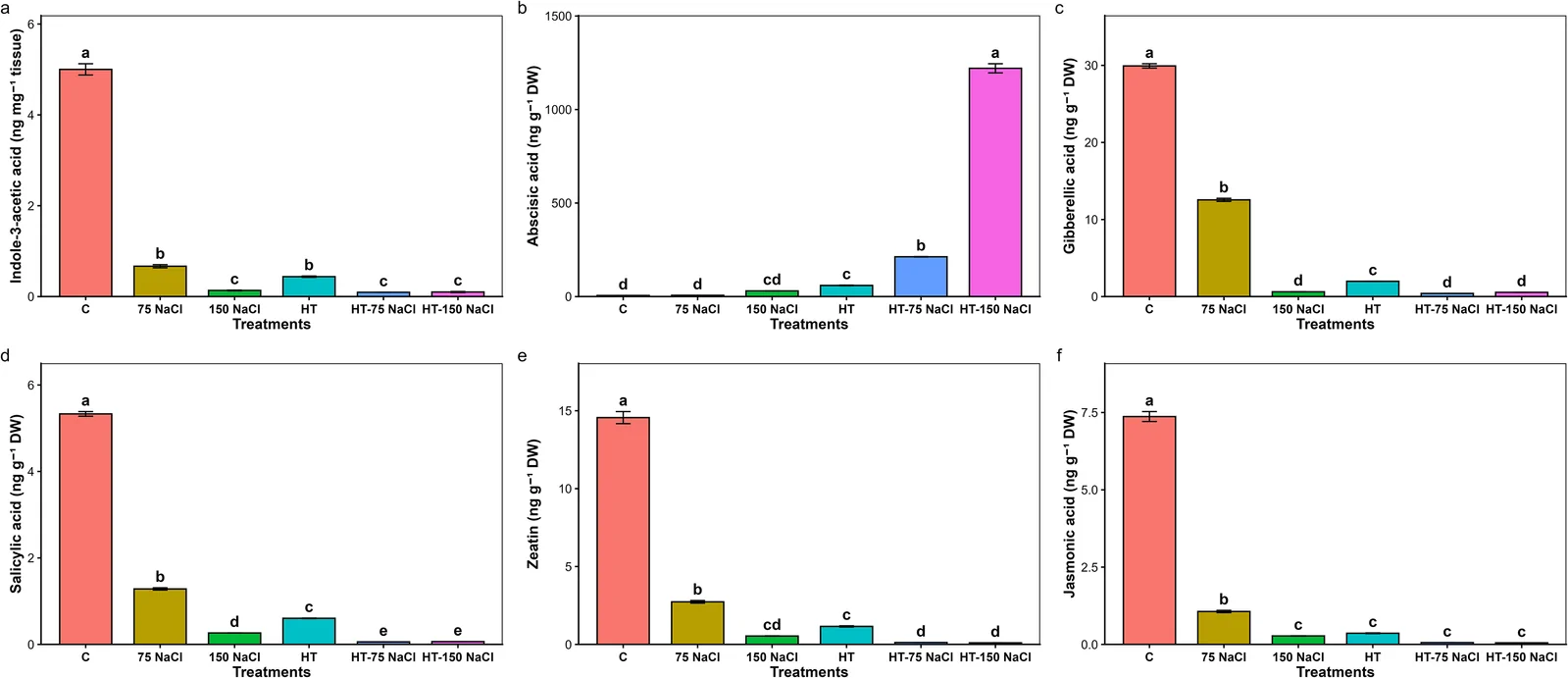
Impact of salinity and high temperature stress on endogenous phytohormone levels, in indole-3-acetic acid **(a)**, abscisic acid **(b)**, gibberellic acid **(c)**, salicylic acid **(d)**, zeatin **(e)**, and jasmonic acid **(f)** in cucumber seedlings. Data is presented as Mean, with standard error represented as error bars. NaCl, sodium chloride; HT, High temperature; C, control. Significant differences are represented by different letters above the bars (*p<0.05*).

### 3.6 Effects on antioxidant enzymes

The antioxidant enzyme activities varied significantly under HT and combined stress conditions, but no differences were found for salinity stress alone compared to control (**Supplementary Table S1**; **Figures 8a–h**). Thus, antioxidant enzymes were not significantly enhanced under single salinity conditions except for peroxidase, which varied significantly under the 150 mM stress condition compared to control (**Figure 8b**).

**Figure 8:**
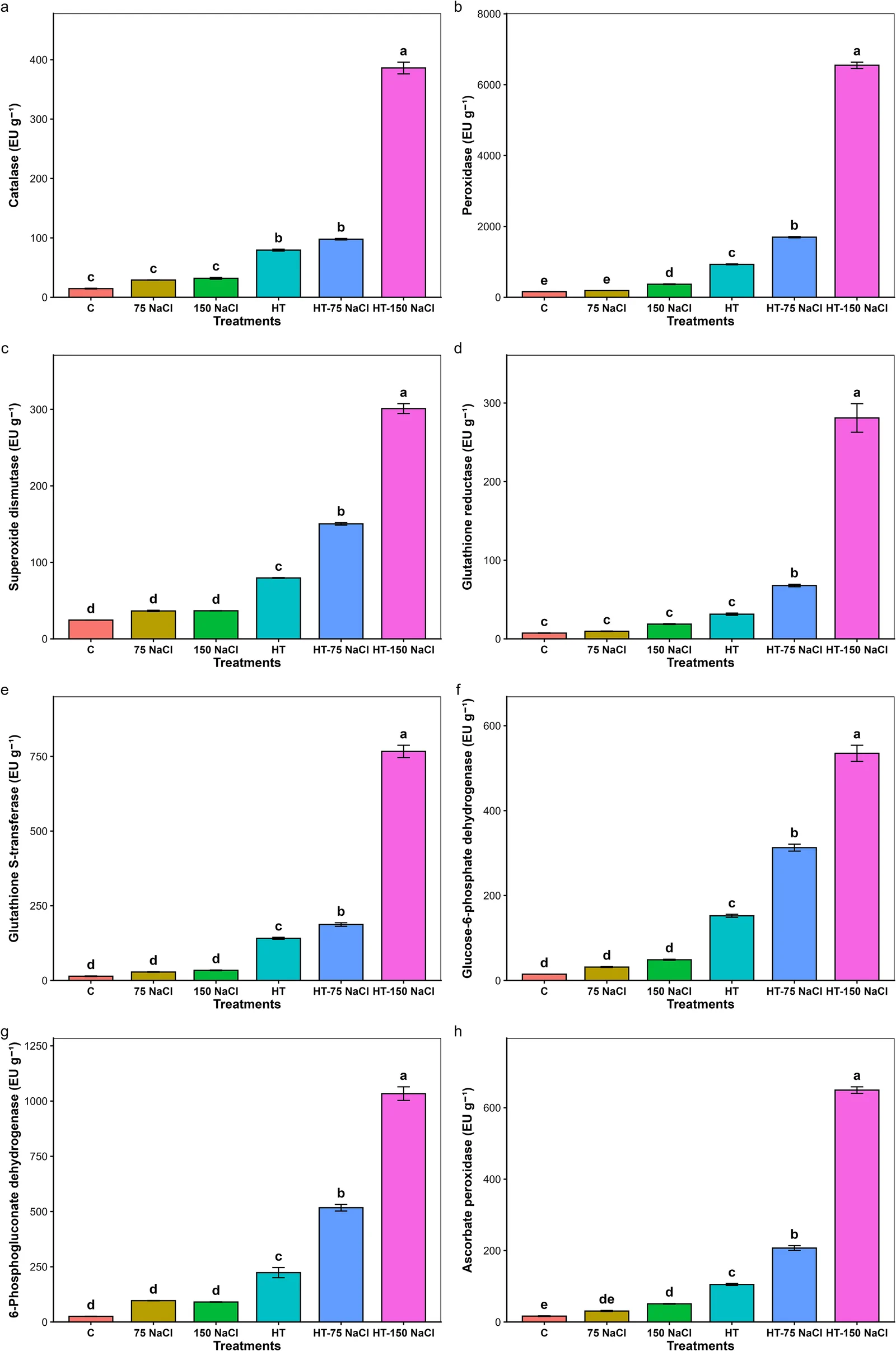
Impact of salinity and high temperature stress on antioxidant enzymes, including catalase **(a)**, peroxidase **(b)**, superoxide dismutase **(c)**, glutathione reductase **(d)**, glutathione S-transferase **(e)**, glucose-6-phosphate dehydrogenase **(f)**, 6-phosphogluconate dehydrogenase **(g)**, and ascorbate peroxidase **(h)** in cucumber seedlings. Data is presented as Mean, with standard errors represented as error bars. NaCl, sodium chloride; HT, High temperature; C, control. Significant differences are represented by different letters above the bars (*p<0.05*).

Under control conditions, the concentrations of CAT, POD, SOD, GR, GST, G6PD, 6PGDH and APX were 14.7, 157.7, 24.6, 7.4, 14.1, 14.7, 25.5 and 16.5 EU g^-1^. However, their activities were enhanced by 98.9, 19.8, 48.6, 31.6, 99.7, and 114.7%, respectively, under 75 mM NaCl stress. 150 mM NaCl stress further aggravated the activities with slight increases of 118.2, 133.9, 49.6, 155.4, 139.4, 232.3, 254.7, and 208.7%, respectively, compared to control conditions. However, the concentrations were significantly increased under HT stress up to 5.4-, 5.9-, 3.2-, 4.2-, 10.0-, 10.4-, 8.8-, and 6.4-fold, respectively, corresponding to 32.0, 368.8, 36.7, 18.8, 33.8, 48.7, 90.6, and 50.9 EU g^-1^, respectively.

Combined stress application resulted in the highest increase in antioxidant enzyme activities, which was drastically more pronounced under HT + 150 mM NaCl stress conditions. Compared to control, HT + 75 mM NaCl increased the activities of CAT, POD, SOD, GR, GST, G6PD, 6PGDH, and APX by 6.7-, 10.8-, 6.1-, 9.2-, 13.3-, 21.3-, 18.5-, and 12.6-fold, respectively. But HT + 150 mM NaCl stress increased these concentrations to 386.1, 6547.9, 301.0, 281.0, 766.7, 535.1, 1033.7, and 649.4 EU g^-1^, respectively, corresponding to an increase of 26.3-, 41.5-, 12.2-, 37.8-, 54.4-, 70.3-, 40.5-, and 39.4-fold, respectively, compared to control conditions.

### 3.7 Association among plant growth and biomass traits, nutrients, hormonal, and antioxidant enzyme responses

Biplot for the principal component analysis (PCA) of all traits under study revealed treatment-dependent responses (**Figure 9a**). PC1 accounted for 75.2% of the variation, while PC2 accounted for 17.1% of the variation, totaling 92.3% of the variation. Control was distinctly separated from the rest of the treatments with strongly positive associations with phytohormones, macro- and micro-nutrients, and morphological and pigment-related traits, reflecting higher values for these traits, while negative associations with antioxidant enzymes and stress-responsive traits indicated the lowest values for these traits. Contrastingly, compound stress of HT + 150 mM NaCl was positioned opposite the control (C) and 75 mM NaCl, indicating a contrasting response of this treatment. Moreover, antioxidant enzymes and stress indicator traits had the highest values for this treatment. All other treatments were relatively clustered in the middle near 75 mM NaCl, indicating moderate values of all the traits under study but relatively lower values for nutrients, hormones, and morphological traits.

**Figure 9:**
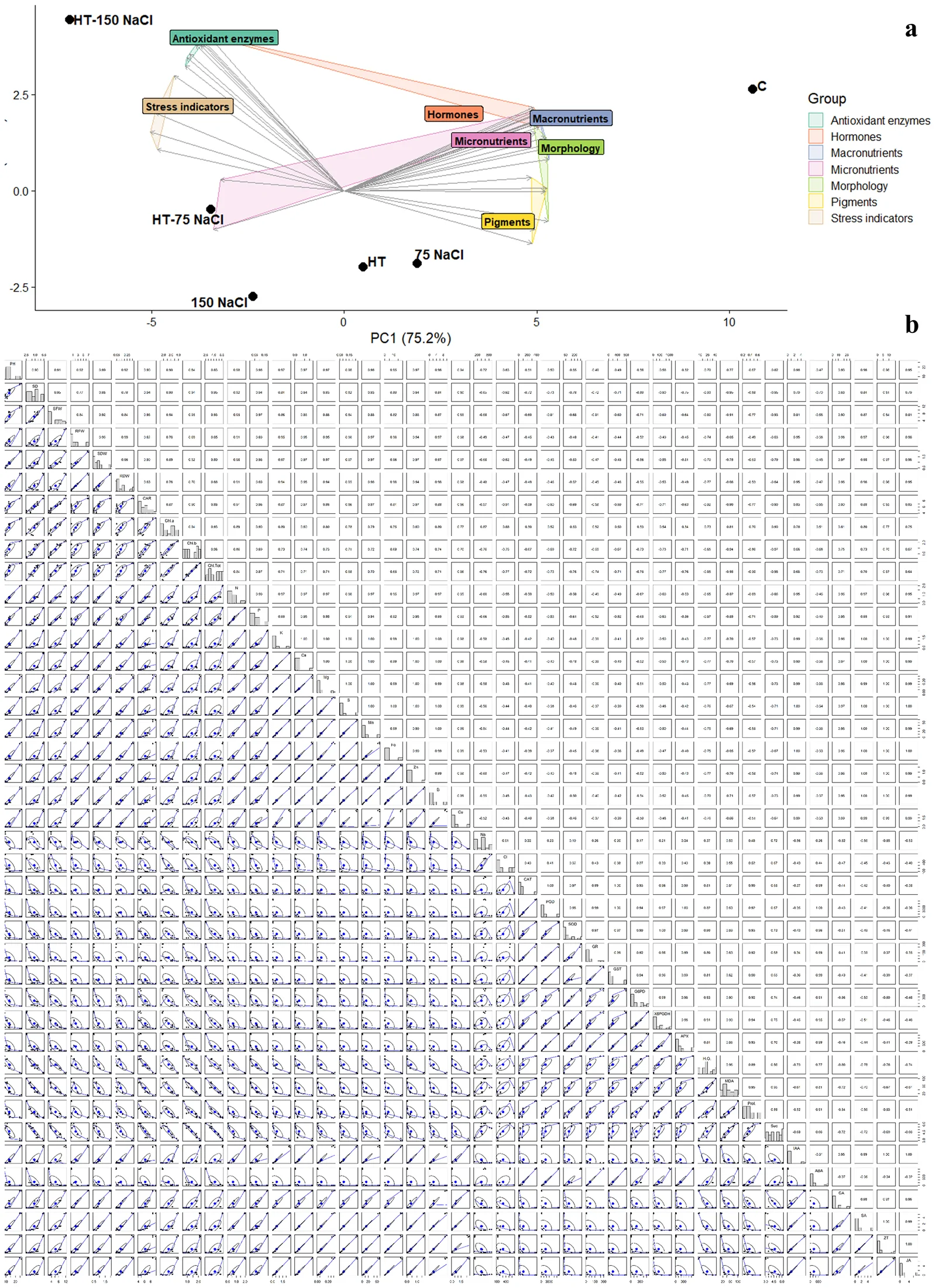
Association among plant growth traits, nutrients, hormonal, and antioxidant enzyme responses in cucumber seedlings. **(a)** Principal Component Analysis biplot presenting distribution of treatments and contribution of traits group including morphology, pigments, macro- and micro-nutrients, phytohormones, stress indicators, and antioxidant enzymes. **(b)** Scatterplot matrix showing Pearson correlations among all the traits under study. The upper panels present Pearson correlation coefficients, the diagonal panels show the distribution of individual variables, and the lower panels display pairwise scatterplots with fitted linear regression lines illustrating the relationships among traits. C, control; NaCl, sodium chloride; HT, High temperature; SD, stem diameter; SFW, shoot fresh weight; RFW, root fresh weight; PH, plant height; SDW, shoot dry weight; RDW, root dry weight; CAR, carotenoids; Chl, chlorophyll; N, nitrogen; K, potassium; P, phosphorus; Ca, calcium; S, sulfur; Mg, magnesium; Fe, iron; Mn, manganese; Zn, zinc; Cu, copper; B, boron; Cl, chloride; Na, sodium; POD, peroxidase; CAT, catalase; SOD, superoxide dismutase; 6PGDH, 6-phosphogluconate dehydrogenase; GR, glutathione reductase; G6PD, glucose-6-phosphate dehydrogenase; GST, glutathione S-transferase; APX, ascorbate peroxidase; MDA, malondialdehyde; H_2_O_2_, hydrogen peroxide; Prol, proline; Suc, sucrose; IAA, indole-3-acetic acid; GA, gibberellic acid; ABA, abscisic acid; ZT, zeatin; SA, salicylic acid; JA, jasmonic acid.

Pearson correlation further strengthened the association among traits (**Figure 9b**). For instance, all the morphological traits showed strong positive associations with each other (r^2^ > 0.9), except for SD, which had ~0.8 values with biomass traits, still significantly positive. Morphological traits were also positively associated with all the nutrient traits (> 0.9) except for the Na and Cl ions, with whom negative association was found (~-0.7). Similarly, nutrients and morphological traits also had a negative association with stress indicators, antioxidant enzymes, and abscisic acid, while having a positive association with the rest of the phytohormones. Stress indicators and antioxidant enzymes had a positive association among themselves.

A heat map was generated to evaluate the cumulative response of all the traits under stress conditions and to cluster the supporting and contrasting traits for each treatment (**Figure 10**). Treatments were categorized into two major groups and three sub-groups, with C (control) as a separate major group, HT + 150 mM NaCl as a secondary major group, while 75 mM NaCl and HT (high temperature) clustered closely together. Similarly, traits were also clustered into two groups, with stress and antioxidant enzymes in one group and rest of the traits in 2nd group. Plant morphological traits, phytohormones, and pigments were clustered together in the sub-group, while Nutrients (micro and macro) were in a separate sub-group. Control and HT + 150 mM NaCl treatments showed contrasting behavior for all the traits. 75 mM NaCl and HT showed similar patterns for the traits.

**Figure 10:**
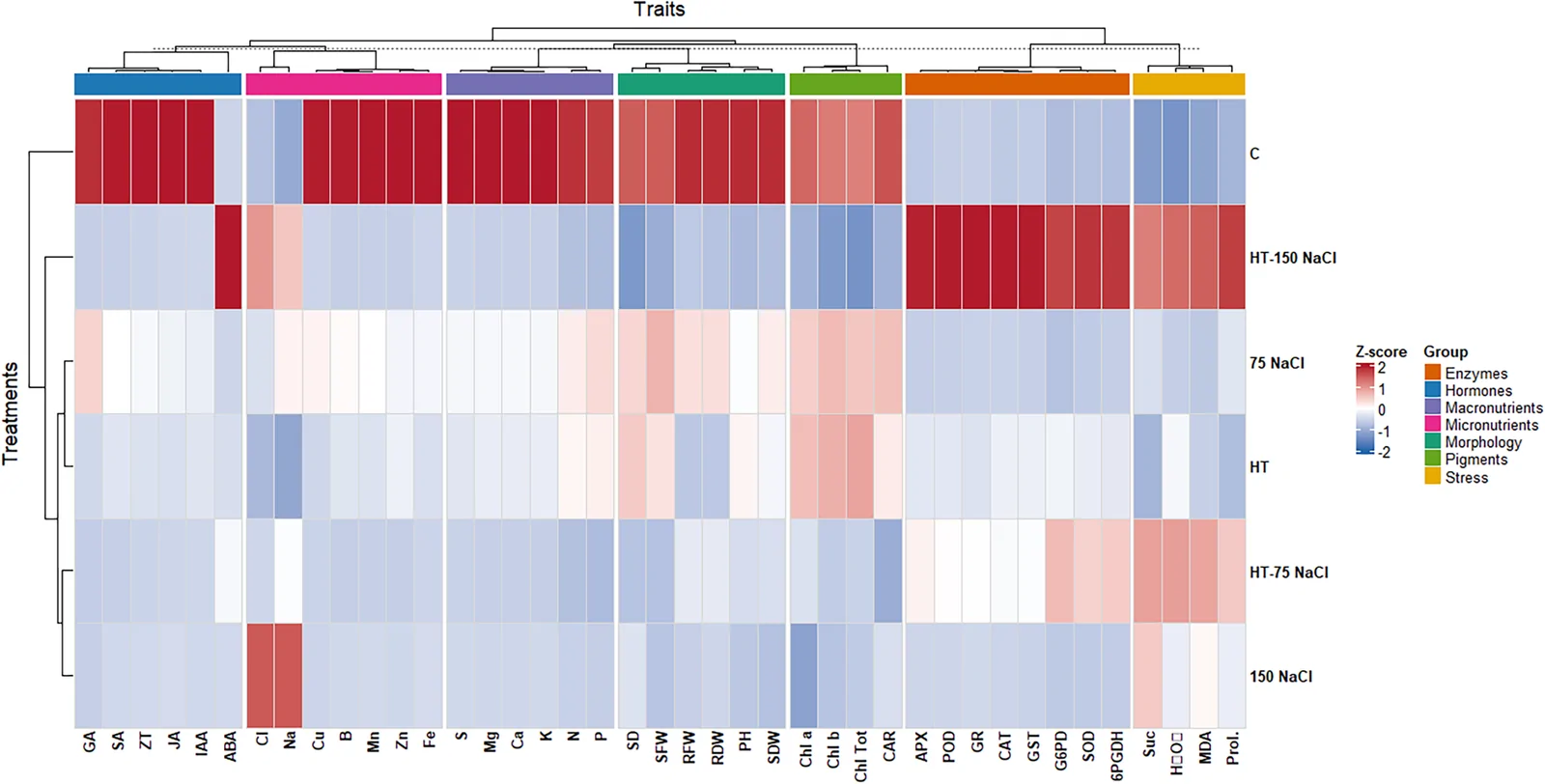
Hierarchical clustering heatmap for the plant growth and biomass parameters, carotenoids and photosynthetic pigments, micro- and macro-nutrients, oxidative stress biomarkers and osmo-protectants, phytohormones, and antioxidant enzymes in cucumber seedlings. Data were standardized using Z-score normalization before clustering. C, control; NaCl, sodium chloride; HT, High temperature; SD, stem diameter; SFW, shoot fresh weight; RFW, root fresh weight; PH, plant height; SDW, shoot dry weight; RDW, root dry weight; CAR, carotenoids; Chl, chlorophyll; N, nitrogen; K, potassium; P, phosphorus; Ca, calcium; S, sulfur; Mg, magnesium; Fe, iron; Mn, manganese; Zn, zinc; Cu, copper; B, boron; Cl, chloride; Na, sodium; POD, peroxidase; CAT, catalase; SOD, superoxide dismutase; 6PGDH, 6-phosphogluconate dehydrogenase; GR, glutathione reductase; G6PD, glucose-6-phosphate dehydrogenase; GST, glutathione S-transferase; APX, ascorbate peroxidase; MDA, malondialdehyde; H_2_O_2_, hydrogen peroxide; Prol, proline; Suc, sucrose; IAA, indole-3-acetic acid; GA, gibberellic acid; ABA, abscisic acid; ZT, zeatin; SA, salicylic acid; JA, jasmonic acid.

## 4 Discussion

Cucumber is considered among the most sensitive crops to climate change, especially heat, drought, and salinity. It is highly sensitive to salt stress, which negatively affects plant growth, fruit yield, and quality (Coşkun et al., 2025). Although it is considered a thermophilic crop with an optimum temperature of 20-30°C and able to tolerate up to 35°C, climate change-driven heat waves (often reaching ≥ 35°C) in cucumber-growing areas damage seedlings, cause oxidative damage, affect protein synthesis, and denature proteins (Zinati and Nazari, 2023). The present study demonstrates the sensitivity of cucumber to high temperature (HT; 40°C) and salinity (75 mM and 150 mM) stresses. Results indicated that while 150 mM salt stress and HT alone disrupted hormonal balance and morpho-physiological traits, the simultaneous occurrence of these stresses substantially disrupted physiological functioning and photosynthetic pigment degradation, reflected in severe biomass losses of cucumber seedlings (**Figures 1**-**8**).

### 4.1 Combined heat and salinity inhibited growth of cucumber seedlings

Salinity and heat stress treatments alone and in combination impaired plant growth (**Figures 1**, **2**). Heat stress drastically reduced root biomass, including root fresh and dry weights (-94 and -86%, respectively; **Figures 1d, f**), preceded by the effects of 150 mM NaCl+HT, with reduction percentages of -93% and 88%, respectively. This suggested that the decrease in root biomass was mainly due to high temperature rather than salinity. Hongal et al. (2023) reported a decrease of 39.4% in PH and 69.4% in SFW of tolerant genotypes under HT stress, while up to 71% reductions were reported in the susceptible group, which are in line with our results depicting a more severe reduction in PH (-44%) and less in SFW (38%). The differential responses of roots and shoots to heat and salinity may reflect differences in the primary physiological targets of these stresses. The greater sensitivity of roots to heat may be associated with impaired root growth, membrane stability, and water and nutrient uptake under elevated temperature. In contrast, the stronger effects of salinity on shoots may result from the combined osmotic and ionic effects of NaCl, including disturbances in ion homeostasis and nutrient balance in aboveground tissues. Under combined stress, increased evaporative demand imposed by high temperature may further interact with the osmotic constraints caused by salinity. Nevertheless, these mechanisms remain hypothetical in the present study because organ-specific ion accumulation, root hydraulic properties, and tissue water status were not directly measured.

Stem diameter, plant height, and shoot biomass were more affected by salinity, with reductions as high as 78% under combined or 150 mM NaCl alone (Figures a–c, e). Previous studies have also reported 50-70% reductions in above-ground canopy biomass under salinity stress (Abdel-Farid et al., 2020; Coşkun et al., 2025). Reduced growth under high salt stress may be due to osmotic stress that restricts water uptake, impairs the photosynthetic machinery (as evident in the current study; **Figure 3**), and diverts energy from growth to stress adaptation (Kumar et al., 2021). These results align with Nahar et al. (2022), who found that heat stress effects were initially more pronounced than salinity stress. Likewise, combined stress had more detrimental effects. However, contrary to our results, no decrease in root growth under HT after prolonged exposure was found. In cucumber at the vegetative stage, increasing salinity reduced shoot length and dry weight, as also reported in the current study (**Figures 1c, e**) (Shaik et al., 2025). Severe growth inhibition observed under combined stresses may be attributed to the interactive effects of osmotic stress, ion toxicity, and heat-induced metabolic disruption (Su et al., 2025). Another influencing factor to consider is the potential effect of changes in a plant’s water metabolism when it is exposed to high temperatures. In this experiment, a control group of plants was kept fully watered throughout the entire process. The researchers watered all experimental plants only according to the control group’s water demand.

### 4.2 Salinity stress impaired photosynthetic machinery more than heat stress

Carotenoids and chlorophyll are essential pigments for photosynthesis. During photosynthesis, carotenoids harvest light and transfer it to chlorophylls via singlet-singlet excitation transfer. Moreover, they also act as antioxidants and photoprotectors against reactive oxygen species (ROS, especially hydrogen, superoxide, and oxygen radicals) produced during photosynthesis. They quench these ROS produced naturally or during any biotic or abiotic stresses in plants (Maoka, 2020), making them important for stress adaptation directly and indirectly. Likewise, chlorophylls are also central not only to photosynthesis but also to stress adaptation mechanisms (Agathokleous et al., 2020). High oxidative stress damages chlorophyll and inhibits its synthesis and accumulation. In the current study, all pigment concentrations were more affected by salinity than by heat stress (**Figure 3**). A previous study in alfalfa evaluating the impacts of heat and salinity also revealed similar patterns, where salt stress reduced chlorophyll concentrations more drastically than control and heat stress treatments (Su et al., 2025). This is in line with the current study, as chlorophyll a concentration under high salt stress was reduced by ~9% compared to combined heat and salinity, and heat stress treatments (**Figure 3b**).

This decline in photosynthetic pigments and carotenoids suggested damage to the photosynthetic machinery, which ultimately translated into poor morphological performance under stress conditions (**Figures 1**, **2**). Coşkun et al. (2025) also concluded that chlorophyll stability is a key determinant of salt stress adaptation in cucumber, as salt stress causes ionic and osmotic stress due to decreased chlorophyll levels, resulting in constrained photosynthesis (Al-Momany and Abu-Romman, 2023).

The pigment responses indicate that the effects of heat and salinity were not entirely stress-specific but also depended on stress intensity. In several cases, heat stress produced pigment reductions comparable to those observed under 75 mM NaCl, whereas the strongest effects were generally observed under 150 mM NaCl and combined heat–salinity stress. Thus, the greater reduction observed at higher salinity levels may reflect a dose-dependent response rather than a simple difference in sensitivity to heat versus salinity.

Changes in Mg content may also relate to the observed chlorophyll responses. Magnesium is the central metal ion in the chlorophyll molecule and is therefore essential for chlorophyll structure and photosynthetic function. In the present study, Mg accumulation decreased under stress conditions, and its overall response pattern was broadly similar to that of chlorophyll pigments. This association suggests that altered Mg content may have contributed to the decline in chlorophyll content under stress. Nevertheless, this interpretation should be considered cautiously because the present study did not directly examine Mg homeostasis and chlorophyll biosynthesis.

### 4.3 High temperature and salinity disrupted nutrient homeostasis and accelerated ion toxicity

Maintaining mineral nutrient levels is fundamental for sustainable photosynthesis, enzyme activation, metabolic regulation, and membrane stability. The present study demonstrated that both salinity and high temperature stress substantially depleted essential nutrients (**Figures 4**, **5**). However, their simultaneous occurrence (even in small doses) almost completely depleted concentrations to <0.06 mg kg^-1^ for all the nutrients. High temperature alone reduced nutrients by 65-89%, maybe attributed partially to reduced root mass. While the reduced root architecture decreases water uptake, the need for evaporative cooling under heat demands more water uptake causing water relations imbalance and thus may also impose water stress as well (Giri et al., 2017). Notably, while high temperature reduced nutrient concentrations comparable to 75 mM NaCl stress, combining heat with salinity resulted in 96.9-98.4% reduction in macronutrients (**Figure 4**) and 98.2-99.6% in micronutrients (**Figure 5**), indicating that salinity stress markedly intensified nutrient depletion. These findings suggested that during combined stress conditions, nutrient homeostasis is a bigger challenge to stabilizing plant metabolism than the expected effects of single stress alone.

Severe declines in N, P, K, Ca, Mg, and S (>79%) under single stress alone reflect impaired physiological processes for nutrient uptake and storage. Under salinity stress, soil water potential decreases, reducing root hydraulic capacity and restricting mass flow and diffusion of nutrients toward the root surface. Consequently, excessive accumulation of Na and Cl ions competitively inhibits uptake of essential ions such as K and Ca because of a shared transport system. Our results also show substantial increases in Na and Cl accumulation of 1405% and 418% (**Figures 5f, g**), respectively, under 150 mM NaCl stress alone. Similar results were reported in Chenopodium, where Na accumulation was 14-fold under salt stress compared with 1.7-fold under heat stress (Shuyskaya et al., 2023). Unlike animals, Na^+^ is a non-essential element in plants, and its excessive accumulation deteriorates plant growth. It causes cytosolic K^+^ efflux (**Figure 4c**), leading to imbalanced nutrient homeostasis, oxidative stress, and nutrient deficiency (Cabot et al., 2014). However, under heat stress, Na and Cl concentrations were reduced by ~20% compared with control (**Figures 5f, g**). Hence, under combined heat and salinity stress, Na and Cl concentrations did not increase as drastically as under 150 mM NaCl stress alone, but were still significantly higher, nonetheless (861 and 315%, respectively). This suggests that nutrient reduction under heat stress may not be due to a shared transport mechanism, but rather due to reduced root growth and biomass (**Figures 1d, f**), which decreases root hydraulic capacity and impairs membrane transport processes. Giri et al. (2017) also demonstrated in tomato plants that decreased nutrient uptake under heat stress is caused by reduced root growth and membrane damage. Moreover, it may also be caused by decreased passive nutrient uptake and root-to-shoot transport through transpiration-driven mass flow, due to a lower transpiration rate under heat stress to conserve water (Giri et al., 2017).

These findings indicated that the severity of combined stress cannot be attributed solely to excessive Na^+^ or Cl^-^ accumulation. Instead, high temperature likely aggravated salinity injury by impairing root growth, membrane integrity, transporter function, and cellular metabolism, thereby reducing the plant’s capacity to acquire both toxic and essential ions (Nahar et al., 2022). The lower Na and Cl accumulation observed under combined heat–salinity stress than under 150 mM NaCl alone should not be interpreted as evidence that ionic toxicity was absent or that nutrient deficiency solely caused growth inhibition. The reduced accumulation of Na and Cl under combined stress may instead reflect impaired root uptake and transport, alter plant water relations, and reduce overall plant growth under high temperature. At the same time, the pronounced reductions in several essential mineral nutrients indicate substantial disruption of mineral nutrition and ion homeostasis. Thus, both nutritional imbalance and ionic effects may have contributed to the growth reduction observed under combined stress, but tissue mineral concentrations alone cannot determine their relative contributions. This experiment clearly shows that plants exposed to high-temperature environments experienced a significant drop in internal nutrient concentration. Among the currently conceivable reasons, in addition to slowed root growth and impaired cell membrane transport functions, part of the reason may be related to the aforementioned changes in the plant’s water state and to alterations in nutrient transport driven by transpiration. However, this experiment had a limitation: the researchers did not directly measure the plants’ transpiration rate, nor did they directly measure the plants’ own water state. Therefore, it is impossible to calculate how large a role the aforementioned mechanism of water and transpiration affecting nutrient concentration played in the observed drop in nutrient concentration.

### 4.4 Combined stress intensified oxidative damage and triggered antioxidant defense system

Under stress conditions like high temperature and salinity, plants produce excessive reactive oxygen species (ROS) as a signaling mechanism to activate the defense system against stress. However, these ROS damage plants’ cellular and metabolic functions. In return, plants overcome this oxidative damage through antioxidant activation via enzymatic and non-enzymatic reactions. The enzymatic components include superoxide dismutase (SOD), catalase (CAT), peroxidase (POD), and ascorbate peroxidase (APX). These antioxidant enzymes carry out the detoxification process of ROS (Kumar et al., 2021). In the current study, oxidative stress markers including proline, hydrogen peroxide, MDA, and sucrose were higher under stress conditions, i.e., 10-772% compared to the control (**Figure 6**). All these indicators were higher under salt stress except for H_2_O_2_ (**Figure 6a**), which was higher under HT stress than under salinity stress alone. The H_2_O_2_ accumulation was 3.1-fold higher under HT stress than control, exceeding that of 150 mM NaCl stress, compared to HT stress alone, which had a 2.9-fold increase. Contrastingly, MDA levels increased ~4.6-fold under 150 mM NaCl stress compared to only ~2.7-fold increase under HT, indicating lipid peroxidation was greater under severe salinity stress. This suggested that HT may have primarily stimulated ROS generation through disruption of photosynthetic electron transport and impairment of photosystem II (Zahra et al., 2023), explaining higher H_2_O_2_ levels (**Figure 6a**) consistent with rapid destruction of chloroplast under HT stress (Liu et al., 2026). However, as photosynthesis was not recorded in current study, further validations may be needed, and these oxidative stress responses cannot be solely attributed to stress without considering hydraulic status of plant. Under HT, relative water content (RWC) was higher (88.26%) compared to 84.4% under control and 86.21% under 150 mM NaCl stress (**Supplementary Figure S1**). It suggested that at this stage, the lipid membrane damage is not as severe as salinity stress evident by lower MDA levels, the leaf may be able to retain its structure and water content under HT stress alone. Whereas salinity caused more extensive membrane damage through prolonged osmotic stress, Na/Cl toxicity, and ionic imbalance, leading to severe lipid peroxidation (Liu et al., 2026).

These contrasting oxidative signatures were also reflected in antioxidant enzyme activities. Despite producing lower membrane damage than severe salinity, HT alone resulted in greater antioxidant enzyme activities (**Figure 8**), with CAT, POD, SOD, GR, GST, G6PD, 6PGDH and APX activities increasing by approximately 148, 152, 117, 67, 317, 213, 147 and 107%, respectively, compared with plants exposed to 150 mM NaCl. This likely indicates that HT stress rapidly activated antioxidant enzyme networks compared to salinity stress and used H2O2 as an early-onset stress signal (Zahra et al., 2023), as was also reported in alfalfa (Giri et al., 2017). Moreover, the interaction between the two stresses was stress-intensifying rather than simply additive. Under HT + 75 mM NaCl stress, antioxidant enzyme activities were enhanced moderately compared to HT alone (23, 83, 89, 116, 33, 105, 132 and 97%, respectively). Under HT + 150 mM NaCl, this increase was 386, 605, 278, 795, 444, 251, 363, and 518%, respectively, compared with HT alone. The pronounced induction of primary ROS scavenging enzymes (CAT, POD, SOD, and APX) as well as glutathione-associated enzymes (GR, GST, G6PD, 6PGDG) demonstrated that cucumber seedlings activated multiple antioxidant mechanisms simultaneously under combined stresses. SOD converts superoxide radicals into H_2_O_2_, which catalase and peroxidase scavenge as the first line of defense against stress. Meanwhile, GR, along with G6PD and 6PGDG, maintains antioxidant enzyme activity by providing reducing power through glutathione redox homeostasis (Noctor et al., 2018). This coordinated, complementary mechanism suggests that severe salinity and HT stress require not only ROS detoxification through scavenging enzymes but also continuous regeneration of non-sacrificial ascorbate and glutathione to support prolonged oxidative stress tolerance.

### 4.5 Osmolyte accumulation and hormonal modulation facilitated stress adaptation

Under stress conditions, proline and soluble sugars are accumulated, as also evident from the current study (**Figures 6c, d**), to protect the cells by maintaining osmotic strength of the cytosol compared to the environment and vacuole. Additionally, proline protects against ROS and supports antioxidant enzymes by stabilizing their structures (Hayat et al., 2012). In the current study, both osmolytes progressively accumulated under stress conditions, especially under salinity stress (~35% higher proline and up to 50% more sucrose compared to HT stress alone). This indicated that osmotic stress caused by salinity was a stronger stimulus for osmolyte accumulation than high-temperature stress (Majidian and Ghorbani, 2024). Under combined stress, however, their concentrations were substantially higher, up to 2.5- 3.0-fold, respectively, under HT + 75 mM NaCl, and 3.4- and 3.7-fold, respectively, under HT + 150 mM NaCl stress compared to the control. Similar results were also reported in alfalfa where combined stress enhanced proline content up to 2.8–11.3 times compared to control (Su et al., 2025). It indicated that HT and osmotic stress caused by salinity acted synergistically to stimulate compatible solute biosynthesis, thereby contributing to osmotic adjustment, stabilization of proteins and membranes, and ROS detoxification.

Phytohormone profiling further verified these patterns by shifting from growth regulation to stress adaptation (**Figure 7**). Both salinity and HT stress suppressed the production of growth-promoting hormones (IAA, GA, ZT; **Figures 7a, c, e**), while promoting ABA accumulation simultaneously (**Figure 7b**). The reduction in IAA was particularly severe during salinity stress, with ~86% and 98% decline under 75 mM and 150 mM salt stress, respectively, compared to HT stress causing a 92% reduction (**Figure 7a**). Similar reductions up to 88-98% were observed for GA across treatments (**Figure 7c**). These reductions in reflected growth hormones are consistent with reduced plant morphological growth (**Figures 1a, b**), as auxins, cytokinin, and gibberellins promote cell division and elongation as well as biomass accumulation (Sosnowski et al., 2023).

Among all phytohormones, ABA increased substantially under combined HT + 150 mM NaCl stress (**Figure 7b**), reaching 212.7 ng g^-1^ under HT + 75 mM NaCl and 1220.5 ng g^-1^ under HT + 150 mM NaCl, representing approximately 36- and 207-fold increases relative to the control, which had only 5.9 ng g^-1^. This antagonistic response of ABA to other growth-promoting hormones has also been reported previously (Wei et al., 2025). This indicates that ABA signaling was synergistically activated only when thermal and ionic stresses occurred simultaneously. Such a response is expected and consistent with the role of ABA in integrating osmotic sensing, regulation, antioxidant defense, and stress-responsive gene expression during compound stresses (Vishwakarma et al., 2017). This is further supported by salicylic acid and jasmonic acid, which showed opposite responses (**Figures 7d, f**). Both hormones declined successively with increasing stress severity, reaching 95-99% under combined stress. These results contrast with the hormonal response reported in rice, where ABA synergistically regulated plant adaptation by interacting with other phytohormones like brassinosteroids (Li et al., 2021). In the current study, however, it was indicated that cucumber used ABA-mediated stress signaling rather than multiple hormones signaling pathways. Such hormonal prioritization could be advantageous because of the central role of ABA in modulating stomatal conductance, osmotic adjustments, and antioxidant activation under dehydration-associated stresses (Das et al., 2025). This is also evident from the increase in ABA concentration under high temperature stress alone without salinity stress, where ABA increased by ~10-fold compared to control and 5-times higher than salinity stress alone. Nonetheless, this increase was substantially lower than both combined treatments showing ~36- and 206-fold increase in concentrations. Higher increase in ABA under HT treatment suggests that heat stress may have imposed a degree of water-related stress, potentially associated with increased evaporation demand or disruption of water uptake and transport (Sadok and Lopez, 2020). Thus, heat stress may have implicated a strong ABA-mediated response in present study. However, as plant water status and transpiration were not directly measured, the possibility of heat-induced water imbalance should be considered as potential explanation rather than definitive mechanism. The concurrent reduction in JA and SA suggests that ABA-dependent signaling becomes the predominant stress-responsive pathway under combined stress, although interactions among these stress-responsive enzymes are complex and context-dependent. However, prolonged suppression of JA and SA pathways may conserve metabolic reserves during stress.

## 5 Conclusion

This study presents the first systematic evaluation of the interactive effects of high temperature and salinity stress on cucumber seedlings, providing new insights into the physiological and biochemical mechanisms underlying their combined effects. Our results demonstrated that simultaneous stress restrained seedling growth and biomass more substantially than either stress alone. Although high salinity alone had the strongest inhibitory effects on plant growth parameters and photosynthetic pigments, the addition of heat stress fundamentally exacerbated the stress response by intensifying nutrient depletion, oxidative stress, and hormonal reprogramming. These results indicated that the detrimental effects of combined stress were not explained solely by the additive effects of each stress, but rather by distinct metabolic interactions. The study also identified that nutrient depletion, rather than accumulation of Na and Cl ions (ionic toxicity), was a more critical determinant of growth inhibition. Overall, salinity was found to be a dominant stressor, causing reductions in plant growth, photosynthetic pigments, nutrient concentrations, and growth-promoting hormones, while high temperature primarily intensified oxidative signaling. Furthermore, both stresses induced distinct oxidative signatures; high temperature promoted H_2_O_2_ accumulation and primed the antioxidant network, whereas salinity caused greater membrane lipid peroxidation and ionic imbalance. Their simultaneous occurrence, however, caused the most severe oxidative damage and triggered a coordinated ROS detoxification mechanism (higher levels of CAT, POD, SOD, and APX) and glutathione-dependent redox homeostasis pathways (GR, GST, G6PD, 6PGDH). Nevertheless, this comprehensive antioxidant response was insufficient to prevent oxidative injury under combined stress. Stress adaptation was characterized by enhanced accumulation of proline and sugars, suppression of growth hormones (IAA, GA, ZT), and a marked induction of ABA, indicating that cucumber plants prioritized osmotic adjustments, redox regulation, and stress signaling over biomass accumulation and growth under combined stress. Multivariate analyses further confirmed that growth reduction was closely associated with nutrient depletion, antioxidant activation, oxidative damage, and ABA induction. These findings advance our understanding of cucumber plant responses to concurrent abiotic stresses and identify nutrient homeostasis, antioxidant metabolism, and ABA signaling as key physiological traits for improving stress resilience. Such knowledge provides practical insights for the sustainable development of salt- and heat-stress-tolerant cucumber cultivars suitable for arid to semi-arid and alkaline-saline soils. Future research should focus on integrating multi-omics technologies to study the molecular response of cucumber under combined stress conditions.

## Data Availability

The raw data supporting the conclusions of this article will be made available by the authors, without undue reservation.

## References

[B1] Abbas F. Faried H. N. Akhtar G. Ullah S. Javed T. Shehzad M. A. . (2023). Cucumber grafting on indigenous cucurbit landraces confers salt tolerance and improves fruit yield by enhancing morpho-physio-biochemical and ionic attributes. Sci. Rep. 13, 21697. doi: 10.1038/s41598-023-48947-z 38066051 PMC10709624

[B2] Abdel-Farid I. Radawy M. Rowezek M. Sheded M. (2020). Effect of Salinity Stress on Growth and Metabolomic Profiling of Cucumis sativus and Solanum lycopersicum. Plants 9, 1626. doi: 10.3390/plants9111626 PMC7700630 33238519

[B3] Agarwal S. Pandey V. (2004). Antioxidant enzyme responses to NaCl stress in *Cassia angustifolia*. Biol. Plant 48, 555–560. doi: 10.1023/B:BIOP.0000047152.07878.e7

[B4] Agathokleous E. Feng Z. Peñuelas J. (2020). Chlorophyll hormesis: Are chlorophylls major components of stress biology in higher plants? Sci. Total Environ. 726, 138637. doi: 10.1016/j.scitotenv.2020.138637 32330746

[B5] Al-Momany B. Abu-Romman S. (2023). Cucumber and salinity. Aust. J. Crop Sci. 17 (7), 581–590. doi: 10.21475/ajcs.23.17.06.p3915

[B6] Angelini R. Manes F. Federico R. (1990). Spatial and functional correlation between diamine-oxidase and peroxidase activities and their dependence upon de-etiolation and wounding in chick-pea stems. Planta 182, 89–96. doi: 10.1007/BF00239989 24197003

[B7] Ayoub N. Ali Z. Sher M. A. Muhammad Abu Bakar S. (2021). Insights into the mechanisms of susceptibility and tolerance against salinity stress in rice plant. Pakistan J. Biochem. Biotechnol. 2, 1–23. doi: 10.52700/pjbb.v2i1.30

[B8] Basu S. Kumar A. Benazir I. Kumar G. J. P. P. (2021). Reassessing the role of ion homeostasis for improving salinity tolerance in crop plants. Physiol. Plant 171, 502–519. doi: 10.1111/ppl.13112 32320060

[B9] Bates L. S. Waldren R. Teare I. (1973). Rapid determination of free proline for water-stress studies. Plant Soil 39, 205–207. doi: 10.1007/BF0001806030311153

[B10] Bremmer J. Mulvaney C. (1996). “ Nitrogen-total,” in Methods of Soil Analysis: Part 2 Chemical and Microbiological Properties, 9.2.2, Second Edition. Ed. Page A. (Madison, Wisconsin: Soil Science Society of America Book Series), 1085–1121.

[B11] Cabot C. Sibole J. V. Barceló J. Poschenrieder C. (2014). Lessons from crop plants struggling with salinity. Plant Sci. 226, 2–13. doi: 10.1016/j.plantsci.2014.04.013 25113445

[B12] Coşkun Ö.F. Aydın A. Toprak S. Başak H. (2025). Rootstock-mediated salinity resilience in cucumber (*Cucumis sativus* L.): integrating physiological traits, genomic stability and machine learning. BMC Plant Biol. 26, 121. doi: 10.1186/s12870-025-07964-y 41413771 PMC12825187

[B13] Das D. Kashtoh H. Panda J. Rustagi S. Mohanta Y. K. Singh N. . (2025). From hormones to harvests: A pathway to strengthening plant resilience for achieving Sustainable Development Goals. Plants (Basel) 14 (15). doi: 10.3390/plants14152322 PMC12348992 40805671

[B14] de Mendiburu F. (2019). agricolae: statistical procedures for agricultural research. R. Package Version 1.3-7 1, 3. Available online at: https://CRAN.R-project.org/package=agricolae (Accessed July 14, 2026).

[B15] Emerton R. Nicolas J. Lombardi A. Di Napoli C. (2026). Global heat stress intensification and its expanding footprint on the human population. Nat. Clim. Change. 16, 989–996. doi: 10.1038/s41558-026-02670-537880705

[B16] Ergin S. Aydogan C. Turhan E. (2021). Physiological effects of high temperature stress in some cucurbit plants. Curr. Trends Natural Sci. 10, 235–245. doi: 10.47068/ctns.2021.v10i19.031

[B17] FAOSTAT (2026). Crop and Livestock Products ( Food and Agriculture Orgamization of the United Nations, Rome, Italy). Available online at: https://www.fao.org/faostat/en/#data/QCL,QC (Accessed July 2026).

[B18] Giri A. Heckathorn S. Mishra S. Krause C. (2017). Heat stress decreases levels of nutrient-uptake and -assimilation proteins in tomato roots. Plants (Basel) 6 (1), 6. doi: 10.3390/plants6010006 28106834 PMC5371765

[B19] Gu Z. Eils R. Schlesner M. (2016). Complex heatmaps reveal patterns and correlations in multidimensional genomic data. Bioinformatics 32 (18), 2847–2849. doi: 10.1093/bioinformatics/btw313 27207943

[B20] Hakeem S. Ali Z. Saddique M. A. B. Habib-Ur-Rahman M. Wiehle M. (2025). Harnessing genetic diversity in wheat to enhance grain nutrition and yield for biofortification breeding. Biol. Res. 58, 33. doi: 10.1186/s40659-025-00606-5 40462198 PMC12135563

[B21] Hassani A. Azapagic A. Shokri N. (2020). Predicting long-term dynamics of soil salinity and sodicity on a global scale. PNAS 117, 33017–33027. doi: 10.1073/pnas.2013771117 33318212 PMC7776813

[B22] Hayat S. Hayat Q. Alyemeni M. N. Wani A. S. Pichtel J. Ahmad A. (2012). Role of proline under changing environments: a review. Plant Signaling Behav. 7, 1456–1466. doi: 10.4161/psb.21949 22951402 PMC3548871

[B23] Hongal D. A. Raju D. Kumar S. Talukdar A. Das A. Kumari K. . (2023). Elucidating the role of key physio-biochemical traits and molecular network conferring heat stress tolerance in cucumber. Front. Plant Sci. 14. doi: 10.3389/fpls.2023.1128928 36895870 PMC9990136

[B24] Hsu P. K. Dubeaux G. Takahashi Y. Schroeder J. I. (2021). Signaling mechanisms in abscisic acid‐mediated stomatal closure. Plant J. 105, 307–321. doi: 10.1111/tpj.15067 33145840 PMC7902384

[B25] Kaur M. Sharma P. (2022). Recent advances in cucumber (Cucumis sativus L.). J. Hortic. Sci. Biotechnol. 97, 3–23. doi: 10.1080/14620316.2021.194595637339054

[B26] Kumar S. Li G. Yang J. Huang X. Ji Q. Liu Z. . (2021). Effect of salt stress on growth, physiological parameters, and ionic concentration of water dropwort (Oenanthe javanica) cultivars. Front. Plant Sci. 12, 660409. doi: 10.3389/fpls.2021.660409 34234795 PMC8256277

[B27] Kuraishi S. Tasaki K. Sakurai N. Sadatoku K. (1991). Changes in levels of cytokinins in etiolated squash seedlings after illumination. Plant Cell Physiol. 32 (5), 585–591. doi: 10.1093/oxfordjournals.pcp.a078120

[B28] Li Q. Xu F. Chen Z. Teng Z. Sun K. Li X. . (2021). Synergistic interplay of ABA and BR signal in regulating plant growth and adaptation. Nat. Plants 7 (8), 1108–1118. doi: 10.1038/s41477-021-00959-1 34226689

[B29] Li Y. Jiang F. He Z. Liu Y. Chen Z. Ottosen C.-O. . (2024a). Higher intensity of salt stress accompanied by heat inhibits stomatal conductance and induces ROS accumulation in tomato plants. Antioxidants 13, 448. doi: 10.3390/antiox13040448 38671895 PMC11047744

[B30] Li Y. Jiang F. Niu L. Wang G. Yin J. Song X. . (2024b). Synergistic regulation at physiological, transcriptional and metabolic levels in tomato plants subjected to a combination of salt and heat stress. Plant J. 117, 1656–1675. doi: 10.1111/tpj.16580 38055844

[B31] Liu R. Wang S. Song J. (2026). New progress in the production, oxidative damage, and scavenging mechanisms of reactive oxygen species in plants under abiotic stress. Front. Plant Sci. 17. doi: 10.3389/fpls.2026.1774033 41868509 PMC13002452

[B32] Lück H. (1965). “ Catalase,” in Methods of Enzymatic Analysis, 1st ed. Ed. Bergmeyer H.-U. ( Academic Press), 885–894. Verlag Chemie G.m.b.H. Germany.

[B33] Majidian P. Ghorbani H. (2024). “ Salinity stress in plants: challenges in view of physiological aspects,” in Abiotic Stress in Crop Plants - Ecophysiological Responses and Molecular Approaches. Eds. Hasanuzzaman M. Nahar K. ( IntechOpen, London).

[B34] Maoka T. (2020). Carotenoids as natural functional pigments. J. Nat. Med. 74, 1–16. doi: 10.1007/s11418-019-01364-x 31588965 PMC6949322

[B35] Nahar L. Aycan M. Hanamata S. Baslam M. Mitsui T. (2022). Impact of single and combined salinity and high-temperature stresses on agro-physiological, biochemical, and transcriptional responses in rice and stress-release. Plants (Basel) 11 (4), 501. doi: 10.3390/plants11040501 35214835 PMC8876766

[B36] Noctor G. Reichheld J.-P. Foyer C. H. (2018). ROS-related redox regulation and signaling in plants. Semin. Cell. Dev. Biol. 80, 3–12. doi: 10.1016/j.semcdb.2017.07.013 28733165

[B37] Olarewaju O. O. Fajinmi O. O. Arthur G. D. Coopoosamy R. M. Naidoo K. (2023). Effect of climate change on the production of Cucurbitaceae species in North African countries. J. Agric. Food Res. 14, 100742. doi: 10.1016/j.jafr.2023.10074242574925

[B38] Ors S. Ekinci M. Turan M. Hakeem S. Yigider E. Aydin M. . (2026). Humic substances enhance waterlogging tolerance in eggplant through differential regulation of redox homeostasis and nutrient acquisition. Horticulturae 12, 974. doi: 10.3390/horticulturae1208097430654563

[B39] Pedersen T. L. (2025). “ Patchwork: the composer of plots,” in R Package Version 1.3.2.9000 Ed.: CRAN (the Comprehensive R Archive Network)). Available online at: https://cran.r-project.org/web/packages/patchwork/index.html.

[B40] Pucciariello C. Banti V. Perata P. (2012). ROS signaling as common element in low oxygen and heat stresses. Plant Physiol. Biochem. 59, 3–10. doi: 10.1016/j.plaphy.2012.02.016 22417734

[B41] Rashidi K. Azizpanah A. Fathi R. Taki M. (2024). Efficiency and sustainability: Evaluating and optimizing energy use and environmental impact in cucumber production. Environ. Sustainability Indic. 22, 100407. doi: 10.1016/j.indic.2024.10040742574925

[B42] Rivero R. M. Mestre T. C. Mittler R. Rubio F. Garcia‐Sanchez F. Martinez V. (2014). The combined effect of salinity and heat reveals a specific physiological, biochemical and molecular response in tomato plants. Plant Cell Environ. 37, 1059–1073. doi: 10.1111/pce.12199 24028172

[B43] Rivero R. M. Mittler R. Blumwald E. Zandalinas S. I. (2022). Developing climate‐resilient crops: improving plant tolerance to stress combination. Plant J. 109, 373–389. doi: 10.1111/tpj.15483 34482588

[B44] Rodrigues F. Sousa B. Soares C. Moreira D. Pereira C. Moutinho-Pereira J. . (2024). Are tomato plants co-exposed to heat and salinity able to ensure a proper carbon metabolism?–An insight into the photosynthetic hub. Plant Physiol. Biochem. 206, 108270. doi: 10.1016/j.plaphy.2023.108270 38091934

[B45] Ryan J. Estefan G. Rashid A. (2001). Soil and Plant Analysis Laboratory Manual (Beirut, Lebanon: International Center for Agricultural Research in the Dry Areas, Aleppo, Syria, National Agricultural Research Center, Islamabad, Pakistan).

[B46] Sadok W. Lopez J. (2020). Transpiration increases under high-temperature stress: Potential mechanisms, trade-offs and prospects for crop resilience in a warming world. Plant Cell Environ. 44, 2102–2116. doi: 10.1111/pce.13970 33278035

[B47] Seneviratne S. I. Zhang X. Adnan M. Badi W. Dereczynski C. Di Luca A. . (2023). “ Weather and climate extreme events in a changing climate,” in Climate Change 2021 – the Physical Science Basis: Working Group I Contribution to the Sixth Assessment Report of the Intergovernmental Panel on Climate Change. Eds. Masson-Delmotte V. Zhai P. Pirani A. Connors S. L. Péan C. Berger S. ( Cambridge University Press, Cambridge, United Kingdom and New York, NY, USA), 1513–1766.

[B48] Shaik A. Karthikeyan R. Kousik C. S. (2025). Growth stage-specific responses of cucumber to salinity stress: germination, seedling establishment, and vegetative development. Front. Plant Sci. 16. doi: 10.3389/fpls.2025.1617809 40880868 PMC12380902

[B49] Shalaby T. A. Taha N. A. Rakha M. T. El-Beltagi H. S. Shehata W. F. Ramadan K. M. A. . (2022). Can grafting manage fusarium wilt disease of cucumber and increase productivity under heat stress? Plants 11, 1147. doi: 10.3390/plants11091147 35567148 PMC9106052

[B50] Shams M. Ekinci M. Ors S. Turan M. Agar G. Kul R. . (2019). Nitric oxide mitigates salt stress effects of pepper seedlings by altering nutrient uptake, enzyme activity and osmolyte accumulation. Physiol. Mol. Biol. Plants 25, 1149–1161. doi: 10.1007/s12298-019-00692-2 31564778 PMC6745581

[B51] Shuyskaya E. Rakhmankulova Z. Prokofieva M. Kazantseva V. Lunkova N. (2023). Impact of salinity, elevated temperature, and their Interaction with the photosynthetic efficiency of halophyte crop *Chenopodium quinoa* willd. Agriculture 13 (6), 1198. doi: 10.3390/agriculture13061198

[B52] Singla P. Bhardwaj R. D. Kaur S. Kaur J. J. (2019). Antioxidant potential of barley genotypes inoculated with five different pathotypes of Puccinia striiformis f. sp. hordei. Physiol. Mol. Biol. Plants 25, 145–157. doi: 10.1007/s12298-018-0614-4 30804637 PMC6352528

[B53] Sosnowski J. Truba M. Vasileva V. (2023). The impact of auxin and cytokinin on the growth and development of selected crops. Agriculture 13, 724. doi: 10.3390/agriculture1303072430654563

[B54] Su L. Huang R. Fu D. Chen Y. Zhang X. Chen Y. . (2025). Effects of combined salt and heat stress on agronomic traits, photosynthetic parameters, and physiological biochemistry in six alfalfa (Medicago sativa L.) cultivars. Plants (Basel) 14 (16), 2479. doi: 10.3390/plants14162479 40872102 PMC12389436

[B55] Sun C. Yu W. Wang Y. Ge W. Zhang M. Xu S. . (2026). Salt tolerance in cucumber: from stage-specific injury and integrative regulation to horticultural deployment. Sci. Hortic. 361, 114860. doi: 10.1016/j.scienta.2026.11486042574925

[B56] Team, R.C (2020). R: A Language and Environment for Statistical Computing, R Foundation for Statistical ( R Foundation for Statistical Computing). Version 4.1.2. doi: 10.1890/0012-9658(2002)083[3097:CFHIWS]2.0.CO;2

[B57] Turan M. Ekinci M. Yildirim Ee. Gunes A. Karagoz K. Kotan R. . (2014). Plant growth-promoting rhizobacteria improved growth, nutrient, and hormone content of cabbage (Brassica oleracea) seedlings. Turkish Journal of Agriculture and Forestry 38 (3), 327–333. doi: 10.3906/tar-1308-62

[B58] Vishwakarma K. Upadhyay N. Kumar N. Yadav G. Singh J. Mishra R. K. . (2017). Abscisic acid signaling and abiotic stress tolerance in plants: a review on current knowledge and future prospects. Front. Plant Sci. 8, 161. doi: 10.3389/fpls.2017.00161 PMC5316533 28265276

[B59] Vu V. Q. (2011). ggbiplot: A grammar of graphics implementation of biplots. R. Package version 0.55, 755. doi: 10.32614/CRAN.package.ggbiplot

[B60] Waqas H. M. Saddique M. A. B. Fiaz S. Alnufaei A. A. Sher M. A. Alharthi B. . (2025). Prediction of RNA editing sites and genome-wide characterization and identification of PERK gene family in Oryza sativa in response to salinity stress. Genet. Resour. Crop Evol. 72, 10317–10340. doi: 10.1007/s10722-025-02565-y30311153

[B61] Wei Y.-S. Javed T. Liu T.-T. Ali A. Gao S.-J. (2025). Mechanisms of Abscisic acid (ABA)-mediated plant defense responses: An updated review. Plant Stress 15, 100724. doi: 10.1016/j.stress.2024.100724

[B62] Wei T. Viliam S. Michael L. Yihui X. Yan J. Jeff Z. . (2021). Corrplot: visualization of a correlation matrix. Package ‘corrplot.’ Statistician 56, e24. doi: 10.32614/CRAN.package.corrplot

[B63] Zahra N. Hafeez M. B. Ghaffar A. Kausar A. Zeidi M. A. Siddique K. H. M. . (2023). Plant photosynthesis under heat stress: effects and management. Environ. Exp. Bot. 206, 105178. doi: 10.1016/j.envexpbot.2022.10517842574925

[B64] Zandalinas S. I. Fritschi F. B. Mittler R. (2021). Global warming, climate change, and environmental pollution: recipe for a multifactorial stress combination disaster. Trends Plant Sci. 26, 588–599. doi: 10.1016/j.tplants.2021.02.011 33745784

[B65] Zandalinas S. I. Peláez‐Vico M.Á. Sinha R. Pascual L. S. Mittler R. (2024). The impact of multifactorial stress combination on plants, crops, and ecosystems: how should we prepare for what comes next? Plant J. 117, 1800–1814. doi: 10.1111/tpj.16557 37996968

[B66] Zinati Z. Nazari L. (2023). Deciphering the molecular basis of abiotic stress response in cucumber (*Cucumis sativus* L.) using RNA-Seq meta-analysis, systems biology, and machine learning approaches. Sci. Rep. 13, 12942. doi: 10.1038/s41598-023-40189-3 37558755 PMC10412635

