# Supplementary material for: Combined heat and salinity stress accelerates oxidative injury and ABA-mediated stress response in cucumber seedlings
Source: Front Plant Sci. 2026 Oct 23;17:1963962. doi: 10.3389/fpls.2026.1963962 (PMC13739490; doi:10.3389/fpls.2026.1963962)
Supplement: Supplementary file 1 [file DataSheet1.docx]

**Combined heat and salinity stress accelerates oxidative injury and ABA-mediated stress response in cucumber seedlings**

**Esma Yigider^1^, Melek Ekinci^2^, Sadia Hakeem^2^, Selda Ors^3^, Murat Aydin^1^, Metin Turan^4^, Ertan Yildirim^2*^**

^1^ Department of Agricultural Biotechnology, Faculty of Agriculture, Atatürk University, Erzurum, Türkiye

^2^ Department of Horticulture, Faculty of Agriculture, Atatürk University, Erzurum, Türkiye

^3^ Department of Agricultural Structures and Irrigation, Faculty of Agriculture, Atatürk University, Erzurum, Türkiye

^4^ Department of Agricultural Trade and Management, Faculty of Business, Yeditepe University, Istanbul, Türkiye

*** Correspondence:** ertanyil@atauni.edu.tr

Realtive water content (%)

| Treatments | RWC |
| --- | --- |
| C | 84.4477 |
| 75 NaCl | 88.4547 |
| 150 NaCl | 86.2135 |
| HT | 88.264 |
| HT-75 NaCl | 80.0334 |
| HT-150 NaCl | 80.9956 |

**Figure S1: Relative water content of above ground portion (leaves and stem) across different treatments. C: control, HT: high temperature, NaCl: sodium chloride/salt stress**

**Table S1: Mean square values for main and interaction effects for all the traits under study**

| Trait | Source | df | SS | MS | F | p-value |
| --- | --- | --- | --- | --- | --- | --- |
| PH | Heat | 1 | 105.125 | 105.125 | 297.730 | <0.001*** |
|  | NaCl | 2 | 374.906 | 187.453 | 530.895 | <0.001*** |
|  | Heat × NaCl | 2 | 88.719 | 44.360 | 125.633 | <0.001*** |
|  | Error | 12 | 4.237 | 0.353 |  |  |
|  | Total | 17 | 572.988 | 33.705 |  |  |
| SD | Heat | 1 | 4.692 | 4.692 | 190.861 | <0.001*** |
|  | NaCl | 2 | 10.291 | 5.146 | 209.309 | <0.001*** |
|  | Heat × NaCl | 2 | 0.023 | 0.012 | 0.476 | 0.632 ns |
|  | Error | 12 | 0.295 | 0.025 |  |  |
|  | Total | 17 | 15.302 | 0.900 |  |  |
| SFW | Heat | 1 | 97.177 | 97.177 | 411.740 | <0.001*** |
|  | NaCl | 2 | 136.267 | 68.134 | 288.697 | <0.001*** |
|  | Heat × NaCl | 2 | 22.029 | 11.014 | 46.688 | <0.001*** |
|  | Error | 12 | 2.832 | 0.236 |  |  |
|  | Total | 17 | 258.305 | 15.194 |  |  |
| RFW | Heat | 1 | 44.199 | 44.199 | 1611.793 | <0.001*** |
|  | NaCl | 2 | 83.239 | 41.620 | 1517.378 | <0.001*** |
|  | Heat × NaCl | 2 | 64.062 | 32.031 | 1167.856 | <0.001*** |
|  | Error | 12 | 0.329 | 0.027 |  |  |
|  | Total | 17 | 191.828 | 11.284 |  |  |
| SDW | Heat | 1 | 1.277 | 1.277 | 680.926 | <0.001*** |
|  | NaCl | 2 | 2.436 | 1.218 | 649.512 | <0.001*** |
|  | Heat × NaCl | 2 | 1.080 | 0.540 | 287.872 | <0.001*** |
|  | Error | 12 | 0.023 | 0.002 |  |  |
|  | Total | 17 | 4.815 | 0.283 |  |  |
| RDW | Heat | 1 | 0.066 | 0.066 | 247.758 | <0.001*** |
|  | NaCl | 2 | 0.130 | 0.065 | 244.258 | <0.001*** |
|  | Heat × NaCl | 2 | 0.059 | 0.030 | 111.375 | <0.001*** |
|  | Error | 12 | 0.003 | 0.000 |  |  |
|  | Total | 17 | 0.259 | 0.015 |  |  |
| CAR | Heat | 1 | 6.778 | 6.778 | 54.986 | <0.001*** |
|  | NaCl | 2 | 12.541 | 6.271 | 50.861 | <0.001*** |
|  | Heat × NaCl | 2 | 1.656 | 0.828 | 6.719 | 0.003** |
|  | Error | 12 | 1.479 | 0.123 |  |  |
|  | Total | 17 | 22.454 | 1.321 |  |  |
| Chl a | Heat | 1 | 10.529 | 10.529 | 36.583 | <0.001*** |
|  | NaCl | 2 | 8.419 | 4.209 | 14.626 | <0.001*** |
|  | Heat × NaCl | 2 | 2.924 | 1.462 | 5.080 | <0.001*** |
|  | Error | 12 | 3.454 | 0.288 |  |  |
|  | Total | 17 | 25.326 | 1.490 |  |  |
| Chl b | Heat | 1 | 2.638 | 2.638 | 31.928 | <0.001*** |
|  | NaCl | 2 | 2.190 | 1.095 | 13.252 | <0.001*** |
|  | Heat × NaCl | 2 | 0.815 | 0.407 | 4.930 | 0.001** |
|  | Error | 12 | 0.991 | 0.083 |  |  |
|  | Total | 17 | 6.634 | 0.390 |  |  |
| Chl Tot | Heat | 1 | 22.226 | 22.226 | 63.676 | <0.001*** |
|  | NaCl | 2 | 20.206 | 10.103 | 28.942 | <0.001*** |
|  | Heat × NaCl | 2 | 6.555 | 3.278 | 9.392 | <0.001*** |
|  | Error | 12 | 4.189 | 0.349 |  |  |
|  | Total | 17 | 53.176 | 3.128 |  |  |
| N | Heat | 1 | 2.238 | 2.238 | 3515.527 | <0.001*** |
|  | NaCl | 2 | 4.688 | 2.344 | 3681.009 | <0.001*** |
|  | Heat × NaCl | 2 | 0.976 | 0.488 | 766.595 | <0.001*** |
|  | Error | 12 | 0.008 | 0.001 |  |  |
|  | Total | 17 | 7.910 | 5.071 |  |  |
| P | Heat | 1 | 0.016 | 0.016 | 69.608 | <0.001*** |
|  | NaCl | 2 | 0.030 | 0.015 | 67.040 | <0.001*** |
|  | Heat × NaCl | 2 | 0.012 | 0.006 | 27.119 | <0.001*** |
|  | Error | 12 | 0.003 | 0.000 |  |  |
|  | Total | 17 | 0.061 | 0.004 |  |  |
| K | Heat | 1 | 0.006 | 0.006 | 206.661 | <0.001*** |
|  | NaCl | 2 | 0.012 | 0.006 | 206.729 | <0.001*** |
|  | Heat × NaCl | 2 | 0.006 | 0.003 | 96.574 | <0.001*** |
|  | Error | 12 | 0.000 | 0.000 |  |  |
|  | Total | 17 | 0.024 | 0.001 |  |  |
| Ca | Heat | 1 | 0.003 | 0.003 | 258.482 | <0.001*** |
|  | NaCl | 2 | 0.006 | 0.003 | 262.188 | <0.001*** |
|  | Heat × NaCl | 2 | 0.003 | 0.001 | 118.873 | <0.001*** |
|  | Error | 12 | 0.000 | 0.000 |  |  |
|  | Total | 17 | 0.011 | 0.001 |  |  |
| Mg | Heat | 1 | 0.001 | 0.001 | 61.325 | <0.001*** |
|  | NaCl | 2 | 0.002 | 0.001 | 51.666 | <0.001*** |
|  | Heat × NaCl | 2 | 0.001 | 0.000 | 24.847 | <0.001*** |
|  | Error | 12 | 0.000 | 0.000 |  |  |
|  | Total | 17 | 0.004 | 0.000 |  |  |
| S | Heat | 1 | 0.002 | 0.002 | 71.592 | <0.001*** |
|  | NaCl | 2 | 0.003 | 0.002 | 60.581 | <0.001*** |
|  | Heat × NaCl | 2 | 0.002 | 0.001 | 36.951 | <0.001*** |
|  | Error | 12 | 0.000 | 0.000 |  |  |
|  | Total | 17 | 0.008 | 0.001 |  |  |
| Mn | Heat | 1 | 0.002 | 0.002 | 82.942 | <0.001*** |
|  | NaCl | 2 | 0.003 | 0.001 | 53.509 | <0.001*** |
|  | Heat × NaCl | 2 | 0.002 | 0.001 | 43.407 | <0.001*** |
|  | Error | 12 | 0.000 | 0.000 |  |  |
|  | Total | 17 | 0.007 | 0.000 |  |  |
| Fe | Heat | 1 | 12300.349 | 12300.349 | 87.645 | <0.001*** |
|  | NaCl | 2 | 21920.056 | 10960.028 | 78.089 | <0.001*** |
|  | Heat × NaCl | 2 | 13630.930 | 6815.465 | 48.535 | <0.001*** |
|  | Error | 12 | 1686.028 | 140.502 |  |  |
|  | Total | 17 | 49537.362 | 2913.963 |  |  |
| Zn | Heat | 1 | 360.663 | 360.663 | 57.978 | <0.001*** |
|  | NaCl | 2 | 765.468 | 382.734 | 61.515 | <0.001*** |
|  | Heat × NaCl | 2 | 558.171 | 279.085 | 44.854 | <0.001*** |
|  | Error | 12 | 74.635 | 6.220 |  |  |
|  | Total | 17 | 1758.938 | 103.467 |  |  |
| B | Heat | 1 | 0.003 | 0.003 | 88.878 | <0.001*** |
|  | NaCl | 2 | 0.005 | 0.003 | 91.204 | <0.001*** |
|  | Heat × NaCl | 2 | 0.004 | 0.002 | 73.488 | <0.001*** |
|  | Error | 12 | 0.000 | 0.000 |  |  |
|  | Total | 17 | 0.012 | 0.001 |  |  |
| Cu | Heat | 1 | 5.675 | 5.675 | 1304.299 | <0.001*** |
|  | NaCl | 2 | 6.077 | 3.039 | 698.426 | <0.001*** |
|  | Heat × NaCl | 2 | 5.712 | 2.856 | 656.474 | <0.001*** |
|  | Error | 12 | 0.052 | 0.004 |  |  |
|  | Total | 17 | 17.516 | 11.574 |  |  |
| Na | Heat | 1 | 1001225.653 | 1001225.653 | 77.124 | <0.001*** |
|  | NaCl | 2 | 1032535.414 | 516267.707 | 39.774 | <0.001*** |
|  | Heat × NaCl | 2 | 241032.208 | 120516.104 | 9.287 | <0.001*** |
|  | Error | 12 | 155834.303 | 12986.192 |  |  |
|  | Total | 17 | 2439627.579 | 143507.505 |  |  |
| Cl | Heat | 1 | 346014.738 | 346014.738 | 103.902 | <0.001*** |
|  | NaCl | 2 | 1163387.752 | 581693.876 | 174.653 | <0.001*** |
|  | Heat × NaCl | 2 | 187038.227 | 93519.114 | 28.077 | <0.001*** |
|  | Error | 12 | 39976.287 | 3331.357 |  |  |
|  | Total | 17 | 1736417.004 | 102142.177 |  |  |
| CAT | Heat | 1 | 20448.333 | 20448.333 | 113.425 | <0.001*** |
|  | NaCl | 2 | 15891.104 | 7945.552 | 44.086 | <0.001*** |
|  | Heat × NaCl | 2 | 11279.783 | 5639.892 | 31.283 | <0.001*** |
|  | Error | 12 | 2163.344 | 180.279 |  |  |
|  | Total | 17 | 49782.563 | 2928.386 |  |  |
| POD | Heat | 1 | 532.890 | 532.890 | 253.188 | <0.001*** |
|  | NaCl | 2 | 796.978 | 398.489 | 189.295 | <0.001*** |
|  | Heat × NaCl | 2 | 375.111 | 187.555 | 89.099 | <0.001*** |
|  | Error | 12 | 25.261 | 2.105 |  |  |
|  | Total | 17 | 1730.238 | 101.779 |  |  |
| SOD | Heat | 1 | 179.783 | 179.783 | 148.154 | <0.001*** |
|  | NaCl | 2 | 252.968 | 126.484 | 104.211 | <0.001*** |
|  | Heat × NaCl | 2 | 97.094 | 48.547 | 40.001 | <0.001*** |
|  | Error | 12 | 14.541 | 1.212 |  |  |
|  | Total | 17 | 544.386 | 32.023 |  |  |
| GR | Heat | 1 | 149.144 | 149.144 | 54.853 | <0.001*** |
|  | NaCl | 2 | 159.516 | 79.758 | 29.328 | <0.001*** |
|  | Heat × NaCl | 2 | 47.559 | 23.780 | 8.745 | <0.001*** |
|  | Error | 12 | 32.630 | 2.719 |  |  |
|  | Total | 17 | 388.849 | 22.873 |  |  |
| GST | Heat | 1 | 104.499 | 104.499 | 160.999 | <0.001*** |
|  | NaCl | 2 | 155.528 | 77.764 | 119.822 | <0.001*** |
|  | Heat × NaCl | 2 | 66.393 | 33.197 | 51.120 | <0.001*** |
|  | Error | 12 | 7.795 | 0.650 |  |  |
|  | Total | 17 | 334.214 | 19.660 |  |  |
| G6PD | Heat | 1 | 113.579 | 113.579 | 107.855 | <0.001*** |
|  | NaCl | 2 | 43.902 | 21.951 | 20.841 | <0.001*** |
|  | Heat × NaCl | 2 | 17.979 | 8.990 | 8.536 | <0.001*** |
|  | Error | 12 | 12.640 | 1.053 |  |  |
|  | Total | 17 | 188.099 | 11.065 |  |  |
| 6PGDH | Heat | 1 | 354.901 | 354.901 | 126.153 | <0.001*** |
|  | NaCl | 2 | 273.622 | 136.811 | 48.639 | <0.001*** |
|  | Heat × NaCl | 2 | 72.826 | 36.413 | 12.946 | <0.001*** |
|  | Error | 12 | 33.759 | 2.813 |  |  |
|  | Total | 17 | 735.107 | 43.242 |  |  |
| APX | Heat | 1 | 2860.383 | 2860.383 | 392.027 | <0.001*** |
|  | NaCl | 2 | 3145.507 | 1572.754 | 215.559 | <0.001*** |
|  | Heat × NaCl | 2 | 1726.432 | 863.216 | 118.301 | <0.001*** |
|  | Error | 12 | 87.574 | 7.298 |  |  |
|  | Total | 17 | 7819.896 | 459.994 |  |  |
| H₂O₂ | Heat | 1 | 12973.432 | 12973.432 | 273.649 | <0.001*** |
|  | NaCl | 2 | 1974.456 | 987.228 | 20.822 | <0.001*** |
|  | Heat × NaCl | 2 | 1061.508 | 530.754 | 11.197 | <0.001*** |
|  | Error | 12 | 568.882 | 47.407 |  |  |
|  | Total | 17 | 16578.277 | 975.193 |  |  |
| MDA | Heat | 1 | 118.658 | 118.658 | 160.717 | <0.001*** |
|  | NaCl | 2 | 28.511 | 14.255 | 19.311 | <0.001*** |
|  | Heat × NaCl | 2 | 20.965 | 10.482 | 14.199 | <0.001*** |
|  | Error | 12 | 8.862 | 0.739 |  |  |
|  | Total | 17 | 177.000 | 10.412 |  |  |
| Prol. | Heat | 1 | 114.109 | 114.109 | 96.096 | <0.001*** |
|  | NaCl | 2 | 128.858 | 64.429 | 54.255 | <0.001*** |
|  | Heat × NaCl | 2 | 43.855 | 21.927 | 18.456 | <0.001*** |
|  | Error | 12 | 14.250 | 1.188 |  |  |
|  | Total | 17 | 301.071 | 17.710 |  |  |
| Suc | Heat | 1 | 4.922 | 4.922 | 28.317 | <0.001*** |
|  | NaCl | 2 | 3.535 | 1.767 | 10.165 | <0.001*** |
|  | Heat × NaCl | 2 | 2.542 | 1.271 | 7.313 | <0.001*** |
|  | Error | 12 | 2.086 | 0.174 |  |  |
|  | Total | 17 | 13.085 | 0.770 |  |  |
| IAA | Heat | 1 | 0.168 | 0.168 | 116.289 | <0.001*** |
|  | NaCl | 2 | 0.328 | 0.164 | 113.568 | <0.001*** |
|  | Heat × NaCl | 2 | 0.163 | 0.082 | 56.587 | <0.001*** |
|  | Error | 12 | 0.017 | 0.001 |  |  |
|  | Total | 17 | 0.677 | 0.040 |  |  |
| ABA | Heat | 1 | 0.001 | 0.001 | 168.167 | <0.001*** |
|  | NaCl | 2 | 0.002 | 0.001 | 169.038 | <0.001*** |
|  | Heat × NaCl | 2 | 0.001 | 0.001 | 87.969 | <0.001*** |
|  | Error | 12 | 0.000 | 0.000 |  |  |
|  | Total | 17 | 0.005 | 0.000 |  |  |
| GA | Heat | 1 | 21.524 | 21.524 | 369.427 | <0.001*** |
|  | NaCl | 2 | 28.115 | 14.057 | 241.358 | <0.001*** |
|  | Heat × NaCl | 2 | 14.068 | 7.034 | 120.779 | <0.001*** |
|  | Error | 12 | 0.699 | 0.058 |  |  |
|  | Total | 17 | 64.406 | 3.789 |  |  |
| SA | Heat | 1 | 20.003 | 20.003 | 519.331 | <0.001*** |
|  | NaCl | 2 | 21.549 | 10.775 | 279.721 | <0.001*** |
|  | Heat × NaCl | 2 | 10.822 | 5.411 | 140.486 | <0.001*** |
|  | Error | 12 | 0.462 | 0.039 |  |  |
|  | Total | 17 | 52.836 | 3.108 |  |  |
| ZT | Heat | 1 | 3.802 | 3.802 | 93.203 | <0.001*** |
|  | NaCl | 2 | 7.112 | 3.556 | 87.155 | <0.001*** |
|  | Heat × NaCl | 2 | 2.944 | 1.472 | 36.081 | <0.001*** |
|  | Error | 12 | 0.490 | 0.041 |  |  |
|  | Total | 17 | 14.347 | 0.844 |  |  |
| JA | Heat | 1 | 0.010 | 0.010 | 72.616 | <0.001*** |
|  | NaCl | 2 | 0.017 | 0.009 | 61.995 | <0.001*** |
|  | Heat × NaCl | 2 | 0.008 | 0.004 | 28.804 | <0.001*** |
|  | Error | 12 | 0.002 | 0.000 |  |  |
|  | Total | 17 | 0.037 | 0.002 |  |  |
